# Rhamnose alleviates the proinflammatory response during endotoxemia via the CEACAM1/LGALS9-p38 axis: Rhamnose alleviates the proinflammatory response

**DOI:** 10.3724/abbs.2025109

**Published:** 2025-07-24

**Authors:** Rongjuan Wei, Tao Zhong, Ke Deng, Xianglong Zhang, Dongping Li, Meiling Chen, Ping Chang, Peng Wu, Zhanguo Liu

**Affiliations:** 1 Department of Critical Care Medicine Zhujiang Hospital Southern Medical University Guangzhou 510000 China; 2 Department of Pathophysiology School of Basic Medical Sciences Southern Medical University Guangzhou 510000 China; 3 Department of Urology Nanfang Hospital Southern Medical University Guangzhou 510000 China

**Keywords:** infection, endotoxemia, rhamnose, CEACAM1, p38

## Abstract

Gut microbiota plays an important role in orchestrating the host immune response. We previously reported that gut microbiota-derived rhamnose enhances the phagocytosis of macrophages, upon which we further asked whether rhamnose has modulatory effects on inflammation. Here, we show that, in an LPS-induced endotoxic mouse model, plasma rhamnose levels are increased. This bacteria-derived sugar alone does not impact inflammatory cytokine homeostasis or cause organ damage. In contrast, it is able to alleviate endotoxin-induced systemic inflammation and organ damage. Mechanistically, in macrophages *in vitro*, rhamnose binds to the V39, D40, and T101 sites of carcinoembryonic antigen-associated cell adhesion molecule 1 (CEACAM1), subsequently promoting the interaction between CEACAM1 and galectin 9 (LGALS9), which increases the protein level of dual-specificity protein phosphatase 1 (DUSP1). This inhibits p38 phosphorylation and thus attenuates the LPS-triggered expressions of proinflammatory factors. Collectively, our results suggest that rhamnose signals via the CEACAM1/LGALS9-p38 axis, which suppresses endotoxemia-associated inflammation, and that rhamnose is a candidate anti-inflammatory agent for the control of infection-induced organ damage.

## Introduction

The infection-induced host uncontrolled inflammatory response and consequent cytokine overproduction are the key drivers of organ damage and failure development [ 1, 2]. For example, during the onset of sepsis, innate immune cells such as macrophages are overactivated by pathogen-associated molecular patterns (PAMPs) and secrete large amounts of cytokines to generate a “cytokine storm”. These inflammatory factors can damage healthy cells and ultimately cause organ dysfunction and failure [ 3– 7]. The intracellular signaling pathways that mediate cytokine production are well known. PAMPs are recognized by pattern recognition receptors (PRRs) in immune cells, triggering downstream signaling cascades [ 8, 9]. Mitogen-activated protein kinase (MAPK) signaling is a well-established proinflammatory pathway, and blocking MAPK activity has been demonstrated to decrease inflammation in various disease contexts [ 10– 12]. Thus, uncovering the upstream regulatory mechanisms of MAPK may provide insights into new pharmacological targets for inflammatory disease therapy.

The gut microbiota has been demonstrated to closely modulate host inflammation [ 13– 15]. On the one hand, bacteria-derived harmful products such as LPS and bacterial DNA could be recognized as PAMPs and directly elicit proinflammatory responses [ 9, 16]. LPS, also referred to as endotoxin, is the most widely used challenge approach to induce infectious inflammation in basic research [17]. On the other hand, it is also evident that the gut microbiota generates various anti-inflammatory compounds, such as short-chain fatty acids and certain secondary bile acids [ 18– 21]. Thus, the detailed role played by the gut microbiota during inflammation progression is complex, and more anti-inflammatory elements need to be explored.

Rhamnose is a bacteria-specific metabolite, which can be synthesized only by the microbiota since host cells do not contain TDP/UDP-rhamnose, the precursor for rhamnose synthesis [ 22, 23]. Studies have shown that rhamnose and polysaccharides with rhamnose residues offer numerous benefits to the host. For example, rhamnose can provide antitumor benefits by slowing tumor cell proliferation and enhancing the immune response [ 24, 25]. Additionally, rhamnose activates the solute carrier protein SLC12A4 to increase macrophage phagocytosis and improve the prognosis of bacterium-infected mice [26]. Furthermore, many natural polysaccharides that contain rhamnose also have a wide range of benefits, such as antiaging and antiobesity effects [ 27, 28]. The immunomodulatory properties of rhamnose are likely responsible for these beneficial effects. However, the contribution of rhamnose to endotoxin-induced systemic inflammation is still largely unknown.

In the present study, we systemically evaluated how rhamnose influences host inflammation during endotoxemia and further elucidated the underlying mechanism involved.

## Materials and Methods

### Animal model

Male-specific pathogen-free C57BL/6 mice, aged 6–8 weeks, were obtained from GemPharmatech (Nanjing, China). The mice were maintained under standard laboratory conditions, which included unrestricted access to food and water and a 12-h light/dark cycle at room temperature. The experimental protocols received approval from the Animal Care and Use Committee of Southern Medical University (approval number: SMUL2021094).

The mice were randomly divided into two groups: the PBS group and the rhamnose treatment group (74 mg/kg administered by gavage, R108982; Aladdin, Shanghai, China). The mice were euthanized 12 h posttreatment, and samples of blood, kidney, lung, and liver were subsequently harvested for further analysis.

For the animal models of endotoxemia, the mice were injected intraperitoneally with LPS (15 mg/kg, L2630; Sigma-Aldrich, St Louis, USA) dissolved in PBS. The mice were administered with 74 mg/kg rhamnose or PBS via oral gavage 2 h prior to intraperitoneal injection of LPS. Survival rates were monitored for 48 h, and the mice were euthanized 12 h after LPS administration to assess systemic inflammation and organ damage. To inhibit carcinoembryonic antigen cellular adhesion molecule (CEACAM1) *in vivo*, the mice received an intraperitoneal injection of anti-CEACAM1 antibody (10 mg/kg, 134534; Biolegend, San Diego, USA) for 1 day as previously described [29]. The subjects were administered with either PBS or rhamnose (74 mg/kg) 2 h prior to the LPS challenge. At 12 h post-LPS challenge, the mice were euthanized, and tissue samples were collected for further analysis.

### Targeted metabolomic analysis

The rhamnose concentration in mouse plasma was analyzed via high-performance liquid chromatography (HPLC). In brief, samples were prepared by centrifugation at 13,400 *g* for 15 min at 4°C. The supernatant was filtered through a 0.22-μm membrane (SLGP0033RB; Merck, Burlington, USA) and used for HPLC detection. The chromatographic separations were conducted using a chromatographic column (Platisil 5 μm NH2, 250 mm × 4.6 mm). The mobile phases were pure water for A and acetonitrile for B, with a gradient elution of 20% A:80% B. The injection volume and flow rate were 50 μL and 1.0 mL/min, respectively.

### Histopathological analysis

Tissues were fixed in 4% paraformaldehyde (PFA) at room temperature, embedded in paraffin and cut into sections (4 μm thick). Organ damage was evaluated via hematoxylin and eosin (HE) staining. HE scores were evaluated in 6 randomly chosen fields per sample as previously described [30]. The change in histological grade on a scale of 0 to 3, 0 was defined as “no finding”, 1 as “mild”, 2 as “moderate”, and 3 as “severe” for each of the parameters. The lung parameters included interalveolar septa thickening, erythrocyte stasis in interstitial and interalveolar spaces, neutrophil infiltration, and alveolar collapse.

### Cell isolation and culture

Murine bone marrow-derived macrophages (BMDMs) were isolated and grown in DMEM supplemented with 10% fetal bovine serum (10099-141; Gibco, Grand Island, USA), 1% penicillin-streptomycin (15140-122; Gibco) and 20 ng/mL macrophage colony-stimulating factor (M-CSF, 416-ML; R&D Systems, Minneapolis, USA). The BMDMs were incubated in a 5% CO _2_ incubator (PU-150A; BOLV, Shanghai, China) at 37°C and matured for further experiments after 7 days. BMDMs were exposed to LPS (100 ng/mL) and treated with 20 μM rhamnose or vehicle at 37°C for 6 h or 15 min to assess mRNA and protein expressions.

THP-1 cells were cultured in RPMI 1640 medium (C118775500BT; Gibco) supplemented with 10% fetal bovine serum and 1% penicillin-streptomycin. THP-1 cells were incubated at 37°C with 5% CO _2_ and differentiated into macrophages (THP-1-dMs) in RPMI 1640 medium by stimulation with phorbol 12-myristate 13-acetate (PMA, 100 ng/mL, S7791; Selleck, Shanghai, China) for 48 h. THP1-dMs were exposed to LPS (500 ng/mL) and treated with either 20 μM rhamnose or vehicle at 37°C for 6 h or 15 min to evaluate mRNA and protein expressions.

### Cell viability assay

To evaluate cell viability, a Cell Counting Kit-8 (CCK-8, GK10001; GLPBIO, Montclair, USA) was used following the manufacturer’s guidelines. In brief, 10 ^5^ cells were placed into each well of 96-well plates, allowed to grow overnight, and subsequently exposed to various concentrations (0, 20, 50, 100, and 500 μM) of rhamnose for 6 h. CCK8 reagent was added to each well, and the mixture was incubated at 37°C with 5% CO _2_ for 1 h. The absorbance was recorded at 450 nm with a SpectraMax M5 microplate reader (Molecular Devices, Sunnyvale, USA).

### Western blot analysis

The cells were lysed via commercial RIPA lysis buffer (89900; Thermo Fisher Scientific, Waltham, USA) supplemented with a protease and phosphatase inhibitor cocktail (P1045; Beyotime, Shanghai, China). Protein quantification was conducted via a BCA assay kit (23225; Thermo Fisher Scientific), and the proteins were then denatured by boiling for 10 min with loading buffer (FD006; Fdbioscience, Hangzhou, China). Proteins were separated by SDS-PAGE and transferred onto a hydrophobic PVDF membrane (IPVH00010; Millipore, Burlington, USA). The membranes were subsequently blocked with blocking buffer (P0252; Beyotime) for 30 min at room temperature and then incubated overnight at 4°C with specific primary antibodies. The membranes were then incubated for 1 h with the corresponding secondary antibodies. The membranes were finally incubated with enhanced chemiluminescence (ECL) solution (P0018AM; Beyotime), and images were captured with a ChemiDoc MP imaging system (Bio-Rad, Hercules, USA). All antibody-related information is displayed in Supplementary Table S1.

### Coomassie blue staining

The post-electrophoresis gel was completely immersed in Coomassie brilliant blue staining solution (P0017; Beyotime) and gently agitated at room temperature for 2 h. The staining solution was then replaced by destaining solution, and the gel was continuously shaken with periodic solution changes until clear background and well-defined protein bands were achieved. After a final rinse with double-distilled water (ddH _2_O), the protein band patterns were analyzed.

### Immunofluorescence microscopy

The localization of rhamnose-binding protein in macrophages was determined via fluorescence microscopy following methods outlined in previous studies [ 26, 31]. A molecular probe was developed using biotinylated rhamnose (Bio-Rha) in conjunction with streptavidin-bound commercial quantum dots (QDs, QS605; Jiayuan, Wuhan, China). The probes were preincubated for 30 min and then added to the THP-1-dMs *in situ* for 30 min to observe the expression of rhamnose-interacting proteins via fluorescence microscopy. For the competitive binding experiment, the cells were initially exposed to free rhamnose for 1 h and then incubated with a rhamnose-QD probe for 30 min, after which the expression of rhamnose-interacting proteins was monitored via a fluorescence microscope (TE2000-S; Nikon, Tokyo, Japan).

### Molecular docking analysis

The CEACAM1 protein crystal structure was retrieved from the Protein Data Bank (ID: 5DZL), and the rhamnose structure was acquired from PubChem. The binding sites between rhamnose and CEACAM1 were analyzed via Schrodinger-Maestro software (version 11.1) as previously described [32]. Proteins and small molecules were pretreated with ‘LigPrep’ and ‘Protein Preparation Wizard’. A grid box was generated via Receptor Grid Generation to encompass the entire binding site and the docking ligand. The docking score was used to evaluate the interactions between rhamnose and CEACAM1.

### Drug affinity responsive target stability (DARTS) experiment

BMDMs and THP-1-dMs were lysed on ice for 30 min in M-PER solution (78501; Thermo Fisher Scientific) supplemented with protease and phosphatase inhibitors. The mixtures were then collected and centrifuged at 15,000 *g* for 15 min to retain the supernatant. Protein was then quantified via the BCA method, and the lysate was adjusted to a uniform protein concentration with 1× TNC buffer (50 mM Tris-HCl, pH 8.0, 50 mM NaCl, and 10 mM CaCl _2_). The diluted lysates were treated with various rhamnose concentrations for 2 h, followed by pronase (10165921001; Roche, Basel, Switzerland) digestion at a 1:1000 (w/w) ratio for 30 min. The lysate was then heated for 10 min by adding 5× loading buffer. To assess the ability of rhamnose to bind to target proteins, Coomassie blue staining or western blotting analyses were performed.

### Cellular thermal shift assay (CETSA)

A CETSA was performed as previously reported [33]. Cells, including BMDMs and THP-1-dMs, were harvested via PBS supplemented with a protease and phosphatase inhibitor cocktail. The cell suspension underwent three freeze-thaw cycles with liquid nitrogen. Next, the cell suspension was centrifuged at 20,000 *g* for 20 min at 4°C to separate the soluble fraction from the cell debris. The lysate was divided into two equal parts. Then, 100 μM rhamnose or an equal volume of PBS was added, and the mixture was incubated for 2 h at room temperature. After incubation, each lysate was further divided into 10 equal parts, heated for 3 min at different temperatures of 37, 41, 44, 47, 50, 53, 56, 59, 63, and 67°C, and then allowed to cool at room temperature for another 3 min. After centrifugation, the supernatant was combined with 5× loading buffer and then analyzed by western blot analysis.

### Transcriptome analysis

RNA was extracted from BMDMs via TRIzol reagent (15596018; Invitrogen, Carlsbad, USA). The RNA was subsequently purified via an RNAClean XP Kit (A63987; Beckman, Brea, USA) and an RNase-Free DNase Set (79254; Qiagen, Hilden, Germany). The purified RNA was used for library preparation and sequencing. RNA sequencing (RNA-seq) was performed via the Illumina NovaSeq 6000 platform. Fastq raw count files were filtered via Seqtk ( https://github.com/lh3/seqtk). The reference genome GRCm38.p4 (mm10) was used for quality control. Stringtie (version: 1.3.0) was used to calculate read counts, and FPKM was used to normalize the read count. Differential expression analysis was performed using edgeR. *P*-values were adjusted for multiple testing using the false discovery rate (FDR) method, and genes with a *q*-value ≤ 0.05 and |fold change| ≥ 2 were defined as differentially expressed genes (DEGs). Gene Ontology (GO) enrichment analysis was subsequently conducted to determine the functional enrichment of the DEGs.

### Quantitative RT-PCR assay

RNA was extracted from tissues or cells via TRIzol reagent (Invitrogen), and cDNA was subsequently synthesized via a cDNA reverse transcription kit (FSQ-101; Toyobo, Osaka, Japan). The transcript levels of the target genes were measured via a 7500 real-time PCR system (Applied Biosystems, Foster City, USA). The relative mRNA expression level was calculated via the 2 ^‒ΔΔCT^ method, with 18S ribosomal RNA as the reference for normalizing and quantifying the target gene expression level. Supplementary Table S2 contains a comprehensive list of all the primers utilized in the study.

### ELISA

IL-6/TNFα protein levels in murine plasma and cell culture supernatants were quantified via commercial ELISA kits (NeoBioscience, Shenzhen, China) according to the manufacturer’s instructions.

### Transient transfection

THP-1-dMs were transfected with CEACAM1 siRNA (CEACAM1-siRNA: 5′-GGACCACAGUCAAGACGAUTT-3′) and LGALS9 siRNA (LGALS9-siRNA: 5′-GCAACACCCAGAUCGACAATT-3′) or control siRNA (NC-siRNA: 5′-UUCUCCGAACGUGUCACGUTT-3′) purchased from GenePharma (Shanghai, China). For overexpression studies, the coding sequence (CDS) of LGALS9-NM_009587-HA was synthesized and cloned separately into the GEYB657 plasmid vector using restriction sites *Nhe*I- *Xba*I. The CDS of CEACAM1-NM_001712-Flag was synthesized and cloned separately into the GEYB657 plasmid vector using restriction sites *Nhe*I- *Eco*RI. The GEYB657 vector elements was arranged as CMV-MCS-EF1A-zsGreen-SV40-Puromycin. All the plasmid vectors were obtained from Geneyuan (Guangzhou, China). Transfection of the siRNAs or overexpression plasmids was performed via Lipofectamine 2000 (Invitrogen) according to the manufacturer’s instructions.

### Immunoprecipitation (IP)

Cells were subjected to LPS (500 ng/mL) and rhamnose (20 μM) treatment for 15 min. Cell lysates were prepared and incubated overnight at 4°C with anti-CEACAM1 or anti-LGALS9 antibodies to immunoprecipitate endogenous proteins. The samples were incubated with protein A/G agarose (P2055; Beyotime) for 3 h at 4°C. The agarose was washed five times with lysis buffer, boiled in 1× SDS loading buffer, and analyzed via western blot analysis. For immunoprecipitation of FLAG- or HA-tagged proteins, cell lysates were incubated with BeyoMag™ Anti-Flag Magnetic Beads (P2115; Beyotime) overnight at 4°C with constant rotation. The supernatant was discarded. After three times wash with TBS, the magnetic beads were adsorbed onto a magnetic frame. Proteins bound to magnetic beads were resuspended in 1× SDS loading buffer, boiled for 5 min, and subsequently analyzed by western blot analysis.

### Statistical analysis

Data analysis and visualization were conducted via GraphPad Prism version 8.3. The experimental results are presented as the mean ± SEM. Statistical comparisons between two groups were performed via two-tailed Student’s *t* test, whereas differences among more than two groups were assessed via one-way analysis of variance (ANOVA). Survival curves were plotted via the log-rank test. *P* < 0.05 was considered to indicate statistical significance.

## Results

### Rhamnose protects against LPS-induced inflammation and organ damage

**Figure 1:**
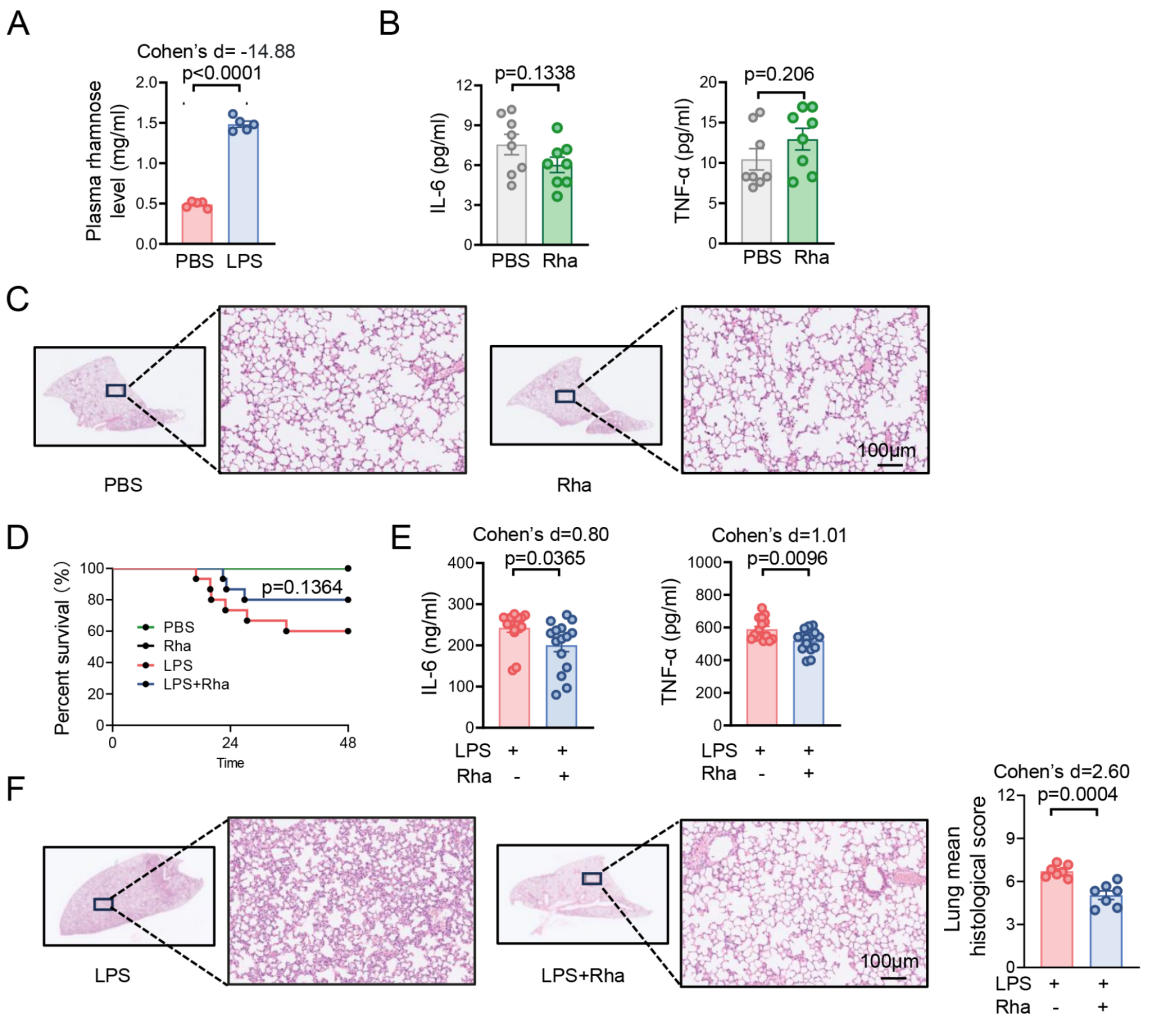
Figure 1 Rhamnose protects against LPS-induced inflammation and organ damage (A) Plasma rhamnose levels. Blood samples were collected 12 h after the intraperitoneal injection of 15 mg/kg LPS or PBS in the mice (n = 5). (B) Plasma IL-6 and TNF-α protein levels. Blood samples were collected 12 h after the oral administration of 74 mg/kg rhamnose or PBS to the mice ( n = 8). (C) Representative HE staining of lung tissues (scale bar = 100 μm). (D) Endotoxemic mice pretreated with rhamnose (74 mg/kg) for 2 h presented improved survival rates (n = 5-15). (E) Plasma IL-6 and TNF-α protein levels in the mice (n = 15). (F) Representative HE staining of lung tissues and corresponding histological scores (n = 7) (scale bar: 100 μm). Effect sizes are reported as Cohen’s d values. Data are shown as the mean ± SEM.

Rhamnose is ubiquitous in bacterial cell walls, and many polysaccharides containing rhamnose have beneficial effects [22]. We first measured plasma rhamnose levels in LPS-induced endotoxemia mice via high-performance liquid chromatography (HPLC). Significantly increased plasma rhamnose levels were observed in mice with LPS-induced endotoxemia ( Figure 1A). We speculated that elevated rhamnose might mediate immune responses to influence infection progression. To address this, we assessed the influence of rhamnose challenge alone under physiological conditions. As shown in Figure 1B,C and Supplementary Figure S1A–C, rhamnose administration neither increased plasma inflammatory factor levels nor caused pathological damage in lung tissues. These findings indicate that rhamnose alone does not induce inflammation-related damage in the physiological state. To explore whether rhamnose affects LPS-induced endotoxemia, we treated mice with rhamnose and then constructed an LPS-induced endotoxemia model. As expected, rhamnose treatment tended to prolong the survival time of LPS-induced endotoxemic mice compared with that of untreated mice ( Figure 1D). Consistently, rhamnose supplementation also substantially attenuated the plasma cytokine levels in the LPS-challenged mice ( Figure 1E). Compared with those in control mice, histological analyses revealed reduced lung damage and limited inflammatory cell infiltration in rhamnose-treated mice ( Figure 1F). Rhamnose decreased inflammatory cytokine and chemokine expression levels in the lung, liver, and kidney tissues of endotoxemic mice ( Supplementary Figure S1D–F). Taken together, these data demonstrate that rhamnose can significantly attenuate LPS-induced organ damage and the inflammatory response.

### Rhamnose inactivates p38 signaling and reduces proinflammatory activity in macrophages, which is associated with increased DUSP1 expression

**Figure 2:**
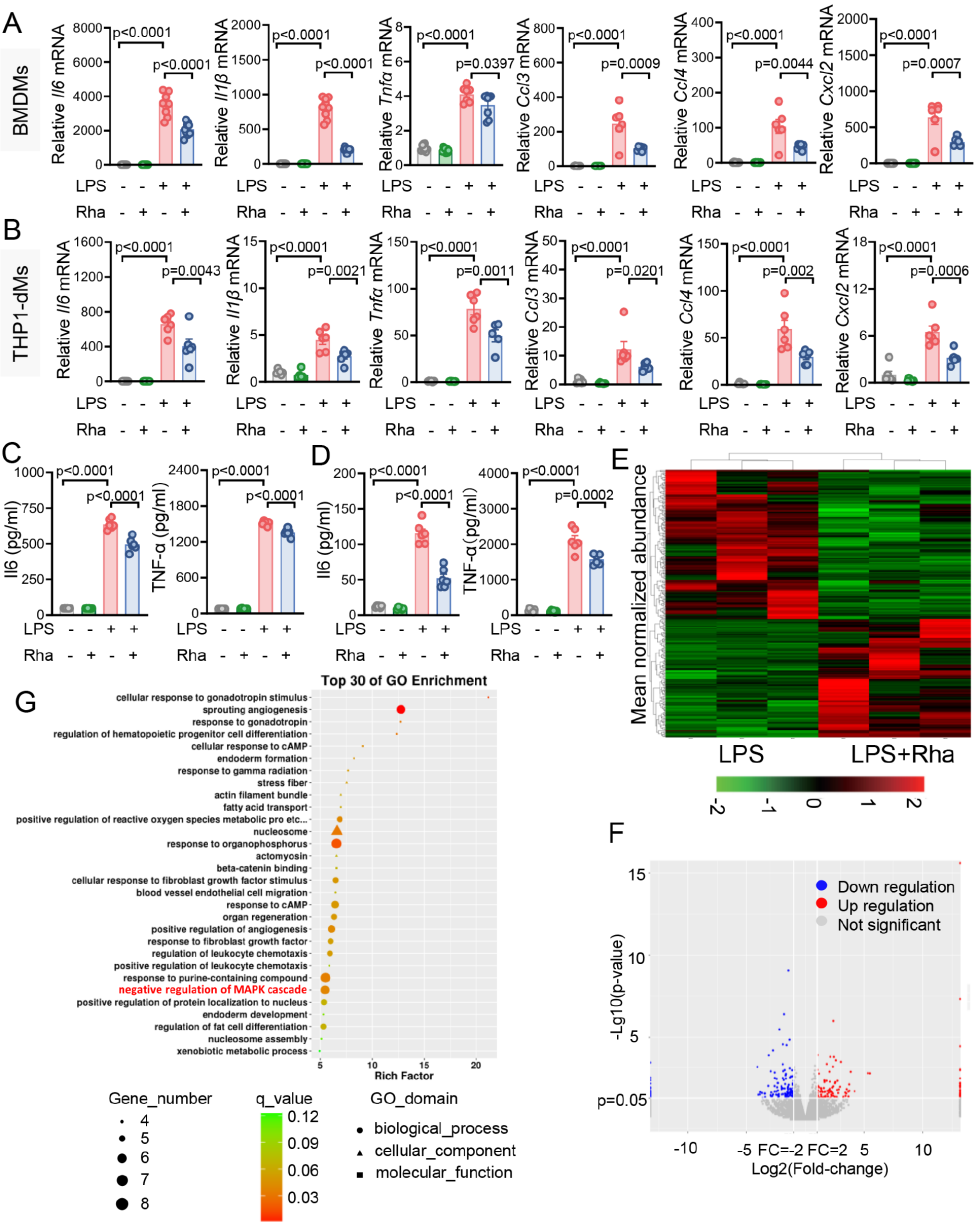
Figure 2 Rhamnose reduces proinflammatory activity in macrophages (A) The mRNA expression of proinflammatory factors was measured in BMDMs following 6 h of LPS (100 ng/mL) stimulation, with or without rhamnose (20 μM) (n = 6–9). (B) The mRNA expression of proinflammatory factors was assessed in THP-1-dMs following 6 h of LPS (500 ng/mL) stimulation, with or without rhamnose (20 μM) (n = 5–6). (C) IL-6 and TNF-α protein levels in the culture supernatant of BMDMs following 6 h of LPS (100 ng/mL) stimulation, with or without rhamnose (20 μM) (n = 6). (D) IL-6 and TNF-α protein levels in the culture supernatant of THP-1-dMs following 6 h of LPS (500 ng/mL) stimulation, with or without rhamnose (20 μM) (n = 6). (E–G) Mature BMDMs were stimulated with LPS (100 ng/mL) for 15 min, with or without rhamnose (20 μM) coincubation. (E,F) Heatmap and volcano plots of RNA-seq data with differential gene cluster analysis (n = 3). Differential expression analysis was conducted using edgeR with FDR correction (q ≤ 0.05). Genes with |fold change| ≥ 2 were defined as differentially expressed genes (DEGs). (G) Gene ontology enrichment of the DEGs (n = 3). DEGs were mapped to GO terms, and a hypergeometric test was used to identify significantly enriched terms compared to the whole genome background. The data are shown as the mean ± SEM.

Next, we explored the underlying mechanism by which rhamnose modulates LPS-mediated systemic inflammation. Given that macrophages significantly contribute to cytokine and chemokine production during the early stages of infection inflammation [ 6, 34 ], we explored the impact of rhamnose on these cells. The anti-inflammatory properties of rhamnose were tested in BMDMs and THP-1-dMs. Consistent with the results of the animal experiments, treating BMDMs and THP-1-dMs with rhamnose alone did not result in any cellular toxicity ( Supplementary Figure S2). After comparing the inflammatory factor levels in the BMDMs and THP-1-dMs, we found that rhamnose treatment significantly reversed the increased levels of proinflammatory factors in the LPS-stimulated macrophages ( Figure 2A–D). These results indicate that rhamnose may target macrophages to control cytokine and chemokine overproduction during inflammatory challenge.

**Figure 3:**
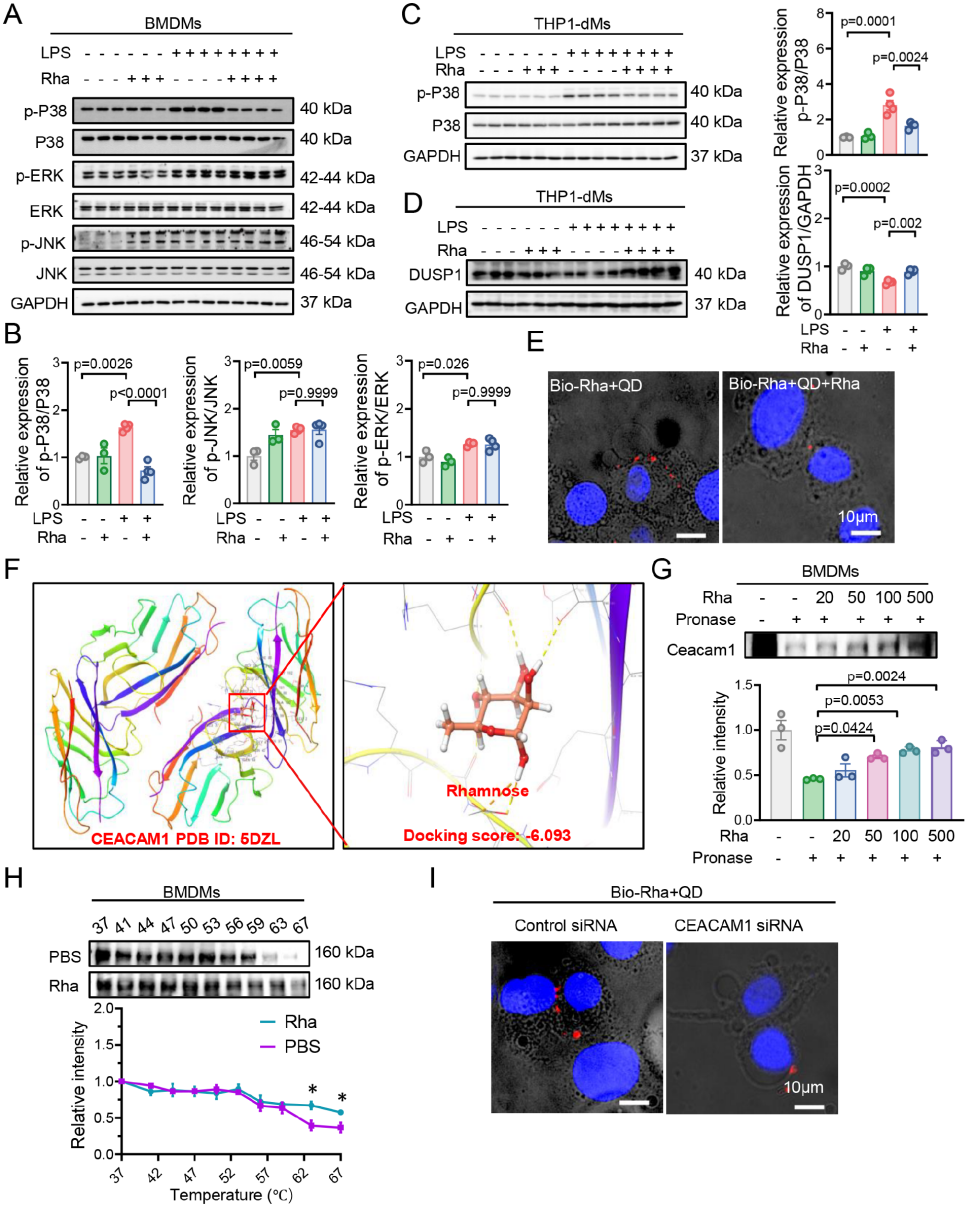
Figure 3 Rhamnose inactivates p38 signal transduction and directly binds to CEACAM1 in macrophages (A,B) Western blot analysis of p-P38, p-ERK, p-JNK, total P38, total ERK, total JNK, and GAPDH levels in BMDMs following 15 min of LPS (100 ng/mL) stimulation, with or without rhamnose (20 μM) (n = 3–4). (C) Western blot analysis of p-P38, total P38, and GAPDH levels in THP-1-dMs following 15 min of LPS (500 ng/mL) stimulation, with or without rhamnose (20 μM) (n = 3–4). (D) Western blot analysis of DUSP1 expression in THP-1-dMs following 15 min of LPS (500 ng/mL) stimulation, with or without rhamnose (20 μM) (n = 3–4). (E) Fluorescence images illustrating biorhamnose (Bio-Rha) binding to THP-1-dMs surfaces (left). Free rhamnose inhibited biorhamnose binding (right). Scale bar = 10 μm. (F) Molecular docking analysis of rhamnose with CEACAM1 (PDB: 5DZL). (G) Western blot analysis of CEACAM1 degradation in pronase-digested lysates from BMDMs with and without rhamnose (n = 3). (H) CETSA analysis of CEACAM1 degradation in BMDM lysates with or without rhamnose (100 μM) treatment for 2 h (n = 3). (I) Fluorescence images illustrating Bio-Rhamnose (Bio-Rha) binding to THP-1-dMs surfaces pretreated with the negative control or CEACAM1 siRNA. Scale bar = 10 μm. The data are shown as the mean ± SEM.

We subsequently analyzed how rhamnose affects macrophages during LPS-induced inflammation via transcriptome profiling of BMDMs. The results revealed that rhamnose administration significantly altered the gene expression profile after LPS exposure ( Figure 2E). Compared with those in the control group (LPS group), 152 genes presented decreased expression, whereas 120 genes presented increased expression ( Figure 2F). GO enrichment analyses of the differentially expressed genes revealed that rhamnose treatment was associated with negative regulation of the MAPK cascade signaling pathway ( Figure 2G). The MAPK signaling pathway is a conserved intracellular signaling system that plays a crucial role in various functions of mammalian cells. This pathway conveys extracellular signals via a series of kinase reactions, resulting in various biological responses, such as cell proliferation, differentiation, apoptosis, and stress responses. In recent years, advancements in molecular biology techniques and extensive research on the MAPK signaling pathway have provided compelling evidence that this pathway plays a significant role in the pathogenesis of inflammatory diseases. Activated MAPK is capable of phosphorylating and activating a variety of transcription factors, notably NF-κB and AP-1, which in turn facilitate the expressions of genes and the synthesis of inflammatory mediators such as TNF-α, IL-1β, and IL-6 [12]. These inflammatory mediators subsequently act on target cells, resulting in the amplification and persistence of the inflammatory response. To further confirm this finding, we examined the impact of rhamnose on the downstream MAPK signaling pathway. As shown in Figure 3A,B, rhamnose significantly inhibited p38 phosphorylation in the MAPK signaling pathway, yet other signals in the pathway were not affected. In addition, the modulation of p38 signaling by rhamnose was reproduced in THP-1-dMs ( Figure 3C). Furthermore, we also used SB203580, a p38 signaling-specific inhibitor, to verify the effects of p38 signaling. In the SB203580 treatment group, rhamnose failed to reduce the expression of p-P38, cytokines, and chemokines after LPS challenge ( Supplementary Figure S3A,B), suggesting that the anti-inflammatory effects of rhamnose are partially mediated through p38 signaling activation.

Given that classical p38 activation is mediated by the MAPKK kinase (MAPKKK)/MAPK kinase (MAPKK) transduction axis [ 35, 36], the p-TAK1 (MAPKKK) and p-MKK3/6 (MAPKK) levels in macrophages after LPS challenge were detected. We found that rhamnose did not influence p-TAK1 or p-MKK3/6 levels ( Supplementary Figure S3C), suggesting that rhamnose may not affect the classical TAK1/MKK3/6/p38 axis. The dual-specificity phosphatase (DUSP) family is a class of protein phosphatases that can remove phosphate groups from MAPK proteins and dephosphorylate other types of proteins. The members of the DUSP family are numerous and can be divided into many different subtypes according to their structure and function. These subtypes differ significantly in their tissue distribution, expression patterns, and regulatory mechanisms, thus endowing the DUSP family with a wide range of regulatory capabilities in cell signaling [37]. During the inflammatory process, dual-specificity phosphatase 1 (DUSP1), an important MAPK phosphatase, has also been shown to preferentially regulate p38 and reduce the production of proinflammatory cytokines and chemokines, leading to an attenuated inflammatory response [38]. On the basis of these findings, we examined the protein levels of DUSP1 and found that, compared with those in the LPS group, the DUSP1 expression in the rhamnose group was significantly upregulated ( Figure 3D). These findings suggest that rhamnose may attenuate the activation of p38 signaling in macrophages, potentially through association with DUSP1.

### Rhamnose directly binds to CEACAM1 in macrophages

To investigate how rhamnose affects the phosphorylation of p38, we synthesized biotinylated rhamnose (Bio-Rha), which binds to streptavidin quantum dots (QDs) as a molecular probe [26], allowing us to monitor the location of the rhamnose-binding protein *in situ*. We first examined its biological effects, as indicated in Supplementary Figure S4A. Biotinylated rhamnose retained its anti-inflammatory properties in THP-1-dMs. After the probes were incubated with the cells for 30 min, the red quantum dots were predominantly located on the cell membrane ( Figure 3E). This finding indicated that the rhamnose-binding protein is located primarily on the cell membrane. Competitive binding experiments were conducted to validate these findings. The cells were incubated with rhamnose for 1 h before binding to the molecular probes, and the immunofluorescence images revealed that rhamnose treatment significantly reduced the red fluorescence on the cell surface ( Figure 3E). These findings verify that rhamnose-binding proteins are predominantly located on the cell surface.

Next, we employed the DARTS assay to screen for proteins that interact with rhamnose. The cell lysate was incubated with either rhamnose or PBS, followed by pronase degradation. Coomassie blue staining revealed that the band at 150–250 kDa was enriched under rhamnose treatment, probably because rhamnose binding resulted in antipronase degradation ( Supplementary Figure S4B,C). A molecular docking/scoring strategy was used for virtual screening to identify the most likely targets of rhamnose ( Figure 3F). Within the expected molecular weight range (150–250 kDa) of membrane proteins associated with inflammation and capable of regulating p38, we focused on CEACAM1. Immunoblot analysis of DARTS samples revealed that rhamnose treatment increased the resistance of the CEACAM1 protein to protease hydrolysis in both BMDMs and THP-1-dMs ( Figure 3G and Supplementary Figure S4D). CETSA also demonstrated that rhamnose reduced the degradation rate of CEACAM1 in heat-denatured BMDMs and THP-1-dMs ( Figure 3H and Supplementary Figure S4E). Additionally, we transfected THP-1-dMs with CEACAM1 or control siRNA and incubated them with biorhamnose-QDs. Immunofluorescence staining revealed a marked reduction in red fluorescence on the cell surface of *CEACAM1*-silenced cells ( Figure 3I), suggesting that CEACAM1 is a target of rhamnose in macrophages.

### CEACAM1 is required in macrophages to suppress the inflammatory response

**Figure 4:**
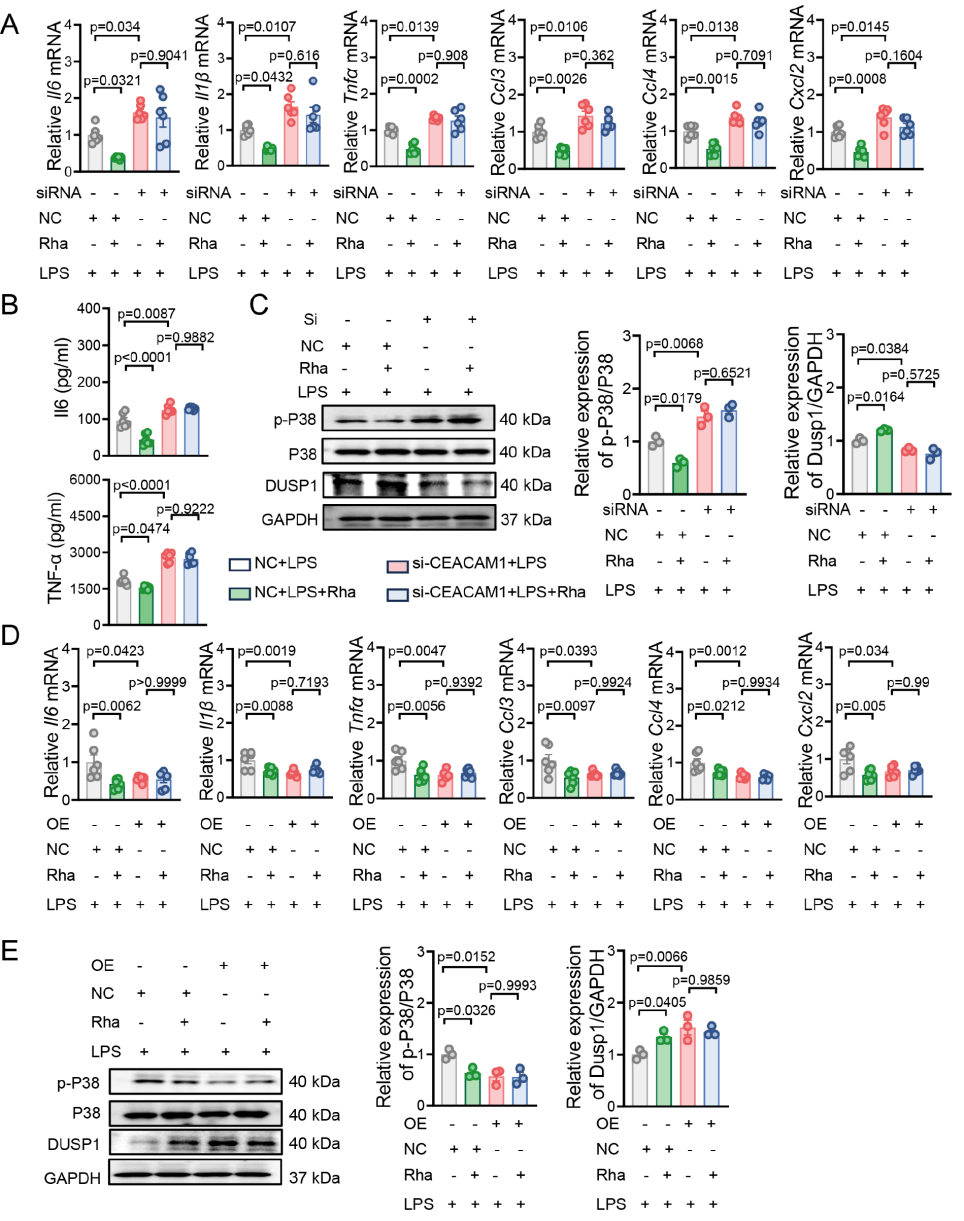
Figure 4 CEACAM1 is required in macrophages to suppress the inflammatory response (A) THP1-dMs were transfected with CEACAM1 or control siRNA for 48 h, followed by a 6-h incubation with LPS (500 ng/mL), with or without rhamnose (20 μM). The mRNA expression levels of major cytokines and chemokines were measured ( n = 6). (B) THP-1-dMs were transfected with CEACAM1 or control siRNA for 48 h, followed by a 6-h incubation with LPS (500 ng/mL), with or without rhamnose (20 μM). The protein levels of IL-6 and TNF-α in the cell culture supernatant were measured (n = 6). (C) THP-1-dMs were transfected with CEACAM1 or control siRNA for 48 h, followed by a 15 min incubation with LPS (500 ng/mL), with or without rhamnose (20 μM). Western blot analysis of p-P38, total P38, DUSP1, and GAPDH levels (n = 3). (D) THP-1-dMs were transfected with empty plasmid or CEACAM1-overexpression plasmid (CEACAM1-OE) for 48 h, followed by a 6-h incubation with LPS (500 ng/mL), with or without rhamnose (20 μM). The mRNA expression levels of major cytokines and chemokines were measured (n = 6). (E) THP1-dMs were transfected with empty plasmid or CEACAM1-overexpression plasmid (CEACAM1-OE) for 48 h, followed by a 6-h incubation with LPS (500 ng/mL), with or without rhamnose (20 μM). Western blot analysis of p-P38, total P38, DUSP1, and GAPDH levels (n = 3). The data are shown as the mean ± SEM.

To evaluate whether rhamnose’s anti-inflammatory effect on macrophages relies on CEACAM1, we employed CEACAM1 siRNA to downregulate its expression in macrophages ( Supplementary Figure S5A,B). The inflammatory response (including increased inflammatory factor levels, decreased DUSP1 expression, and increased p-P38 expression) resulted from LPS activation was significantly enhanced after *CEACAM1* knockdown, suggesting the key status of CEACAM1 in regulating inflammatory activities in macrophages ( Figure 4A–C). Notably, rhamnose had little effect on the group with silenced *CEACAM1* ( Figure 4A–C). Furthermore, we transfected THP-1-dMs with a CEACAM1 overexpression plasmid (OE-CEACAM1) ( Supplementary Figure S5C,D). Upregulated CEACAM1 level significantly decreased inflammatory factor levels, increased DUSP1 expression, and decreased p-P38 expression, which could not be further altered by rhamnose treatment ( Figure 4D,E). Thus, rhamnose may inhibit the LPS-induced excessive inflammatory response in macrophages via CEACAM1.

### Rhamnose promotes the binding of CEACAM1 to LGALS9

**Figure 5:**
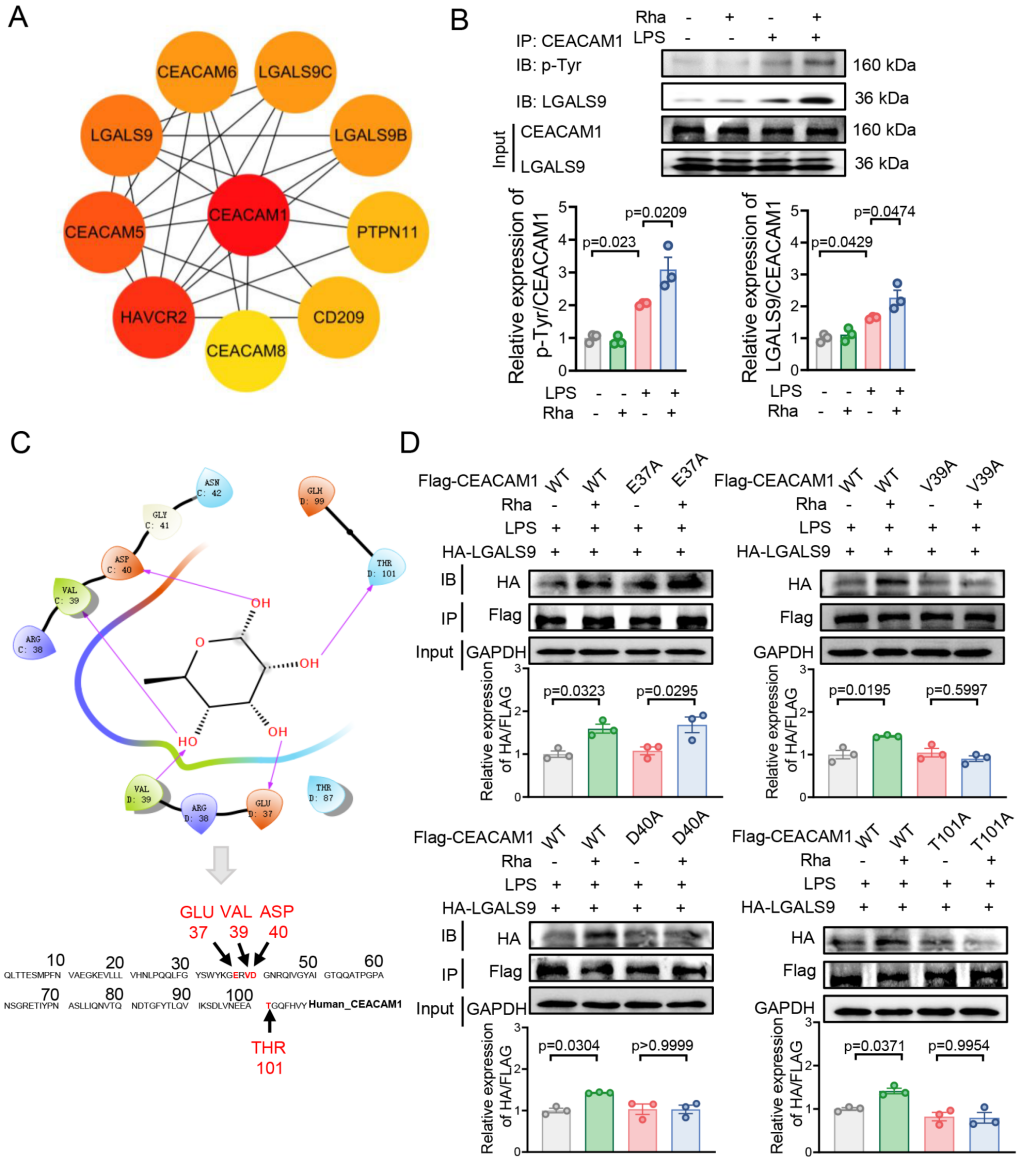
Figure 5 Rhamnose promotes the binding of CEACAM1 to LGALS9 (A) PPI network of CEACAM1-related targets from the STRING database (nodes with a darker color have a greater degree value). (B) Western blot analysis of p-Tyr and LGALS9 in THP-1-dMs exposed to LPS (500 ng/mL) for 15 min, with or without rhamnose (20 μM) (n = 3). (C) Potential interaction binding sites of rhamnose with CEACAM1. (D) THP1-dMs were cotransfected with HA-LGALS9 and different Flag-CEACAM1 mutants, with or without rhamnose (20 μM) treatment. Immunoprecipitation (IP) was performed using an anti-Flag antibody, followed by western blot analysis to assess the effect of rhamnose treatment on the binding of CEACAM1 to LGALS9 (n = 3). The data are shown as the mean ± SEM.

To further explore CEACAM1 downstream signaling mediated by rhamnose, we used the STRING database to mine the key downstream targets of CEACAM1. We focused on LGALS9 because it may interact with CEACAM1, as indicated by our screening via the STRING database ( Figure 5A), and is involved in the inflammatory response of macrophages [39]. These findings led us to speculate that rhamnose might regulate the inflammatory response through the binding of CEACAM1 to LGALS9. To prove that LGALS9 is a potential target of CEACAM1, an immunoprecipitation (IP) experiment was conducted. As expected, the IP assays revealed that CEACAM1 was physically related to LGALS9 ( Figure 5B and Supplementary Figure S6).

Notably, *CEACAM1* encodes two immunotyrosine-based inhibitory motifs (ITIMs) that, when phosphorylated, endow CEACAM1 with inhibitory functions in macrophages, T cells, neutrophils, and other immune cells [ 40, 41]. Research has indicated that LPS treatment induces tyrosine phosphorylation of CEACAM1 in bone marrow-derived monocytes, which subsequently modulates downstream signaling molecules to exert an anti-inflammatory effect [42]. The tyrosine phosphorylation of CEACAM1 suppresses Toll-like receptor 2-triggered antibacterial responses in human lung epithelial cells [43]. These findings suggest that the tyrosine phosphorylation of CEACAM1 significantly impacts its function, particularly in signal transduction and the regulation of immune responses. Given that CEACAM1 can bind to LGALS9, we hypothesized that rhamnose may enhance the tyrosine phosphorylation of CEACAM1 following LPS stimulation, facilitating the interaction with LGALS9 and subsequently increasing DUSP1 expression. To evaluate this, we immunoprecipitated (IP) CEACAM1 in LPS-treated macrophages, and western blot analysis was performed with anti-phosphotyrosine and LGALS9 antibodies. Significant amounts of tyrosine-phosphorylated CEACAM1 and LGALS9 were coimmunoprecipitated in LPS-treated macrophages, and this effect was more pronounced after rhamnose treatment ( Figure 5B). These findings suggest that CEACAM1 may undergo tyrosine phosphorylation at its ITIMs during rhamnose-mediated anti-inflammatory processes and that LGALS9 is physically associated with CEACAM1.

Molecular docking analysis revealed four key amino acid residues (E37A, V39A, D40A, and T101A) in the CEACAM1 protein’s active site that can interact with rhamnose ( Figure 5C). To further confirm whether these predicted sites contribute to rhamnose promotion of CEACMM1 and LGALS9 incorporation, THP1-dMs were transfected with Flag-tagged WT or mutant CEACAM1 plasmids (E37A, V39A, D40A, or T101A) and HA-tagged LGALS9, with or without rhamnose treatment. As indicated in Figure 5D, rhamnose failed to promote the binding of CEACAM1 to LGALS9 after the mutations at the V39A, D40A, or T101A site of CEACAM1, implying that rhamnose may functionally bind to CEACAM1 at the V39A, D40A, and T101A sites (but not E37A) and further affect its interaction with LGALS9. As previously mentioned, overexpression of WT CEACAM1 significantly reduced downstream p-P38 expression, with rhamnose having no further impact on this effect ( Figure 4E and Supplementary Figure S7). Interestingly, overexpression of the E37A mutant CEACAM1 retained its protein activity, whereas overexpression of the V39A, D40A, and T101A mutant CEACAM1s impaired its function, abolishing their inhibitory effect on downstream p-P38 ( Supplementary Figure S7).

### Rhamnose inhibits LPS-induced inflammation in an LGALS9-dependent manner

**Figure 6:**
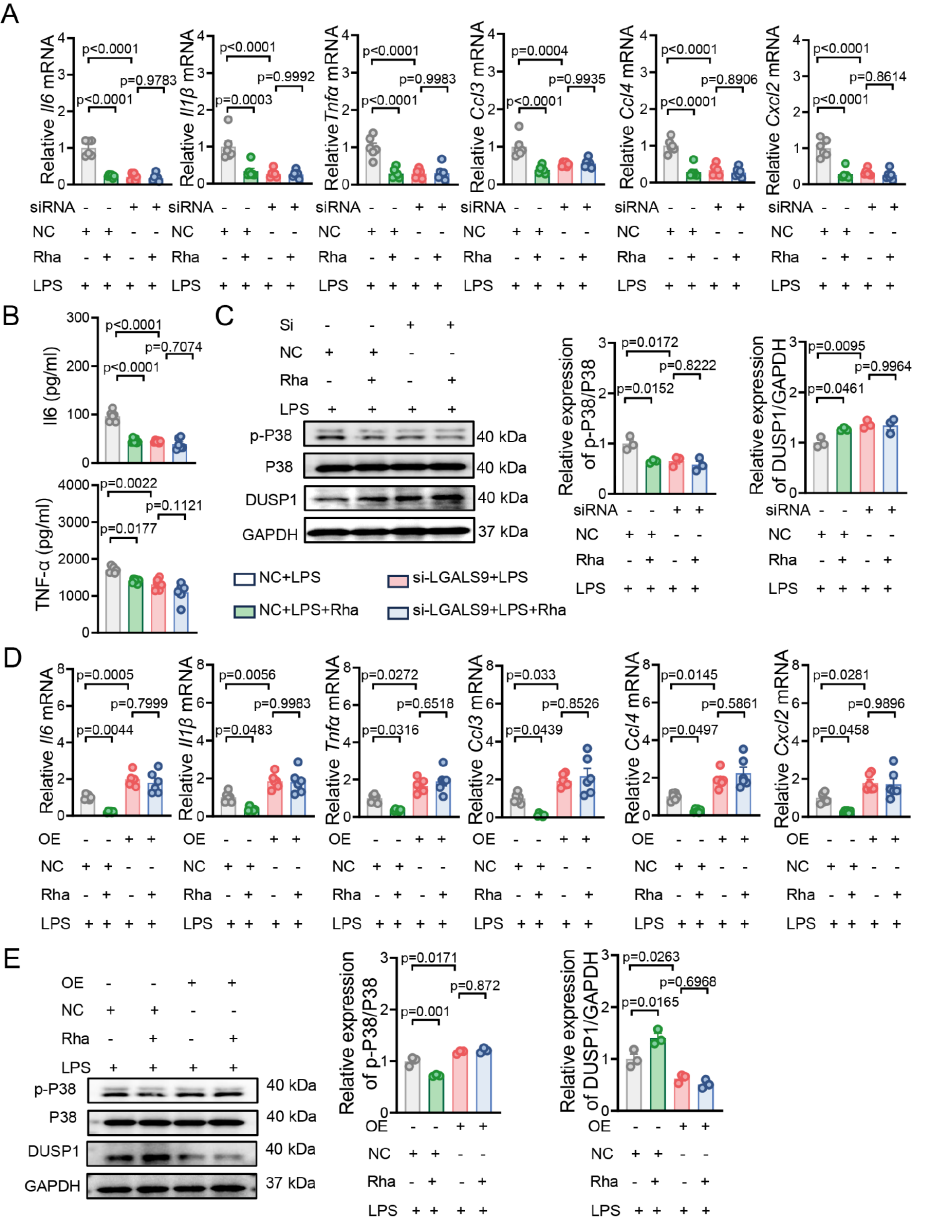
Figure 6 Rhamnose inhibits LPS-induced inflammation in an LGALS9-dependent manner (A) THP-1-dMs were transfected with LGALS9 or control siRNA for 48 h, followed by a 6-h incubation with LPS (500 ng/mL), with or without rhamnose (20 μM). The mRNA levels of major cytokines and chemokines were measured (n = 6). (B) THP-1-dMs were transfected with LGALS9 or control siRNA for 48 h, followed by a 6-h incubation with LPS (500 ng/mL), with or without rhamnose (20 μM). The protein levels of IL-6 and TNF-α were measured in the cell culture supernatant (n = 6). (C) THP1-dMs were transfected with LGALS9 or control siRNA for 48 h, followed by a 15 min incubation with LPS (500 ng/mL), with or without rhamnose (20 μM). Western blot analysis of p-P38, total P38, DUSP1, and GAPDH levels ( n = 3). (D) THP1-dMs were transfected with empty plasmid or LGALS9-overexpression plasmid (LGALS9-OE) for 48 h, followed by a 6-h incubation with LPS (500 ng/mL), with or without rhamnose (20 μM). The mRNA levels of major cytokines and chemokines were measured (n = 6). (E) THP1-dMs were transfected with empty plasmid or LGALS9-overexpression plasmid (LGALS9-OE) for 48 h, followed by a 15 min incubation with LPS (500 ng/mL), with or without rhamnose (20 μM). Western blot analysis of p-P38, total P38, DUSP1, and GAPDH levels (n = 3). The data are shown as the mean ± SEM.

To further validate the LGALS9-dependent effects of rhamnose on the inflammatory response in macrophages, cells were transfected with siRNA-LGALS9 ( Supplementary Figure S8A,B) and then treated with PBS or rhamnose. As indicated in Figure 6A–C, *LGALS9* silencing markedly increased DUSP1 expression, inactivated p38, and reduced inflammatory factor levels, indicating that LGALS9 is a critical proinflammatory molecule in macrophages. Importantly, rhamnose had limited anti-inflammatory effects in the *LGALS9*-silenced groups, as evidenced by comparable DUSP1, p-P38, and inflammatory factor expression between the rhamnose-treated and untreated groups ( Figure 6A–C). Next, we transfected THP-1-dMs with an LGALS9-overexpressing (OE-LGALS9) plasmid ( Supplementary Figure S8C,D). In contrast, upregulation of LGALS9 markedly diminished DUSP1 expression, activated p38, and increased inflammatory factor levels, which could not be further altered by rhamnose treatment ( Figure 6D,E). Collectively, these data indicate that LGALS9 may serve as a proinflammatory regulatory molecule and that rhamnose-mediated restriction of the excessive inflammatory response in macrophages may be partially dependent on LGALS9. Notably, CEACAM1 had no effect on LGALS9 expression ( Supplementary Figure S9A,B). Therefore, we speculate that CEACAM1 inhibits the LPS-induced inflammatory response possibly by binding to LGALS9 and suppressing its activity without affecting LGALS9 expression.

### CEACAM1 signaling is required for rhamnose anti-inflammatory effects in LPS-induced endotoxemia mice

**Figure 7:**
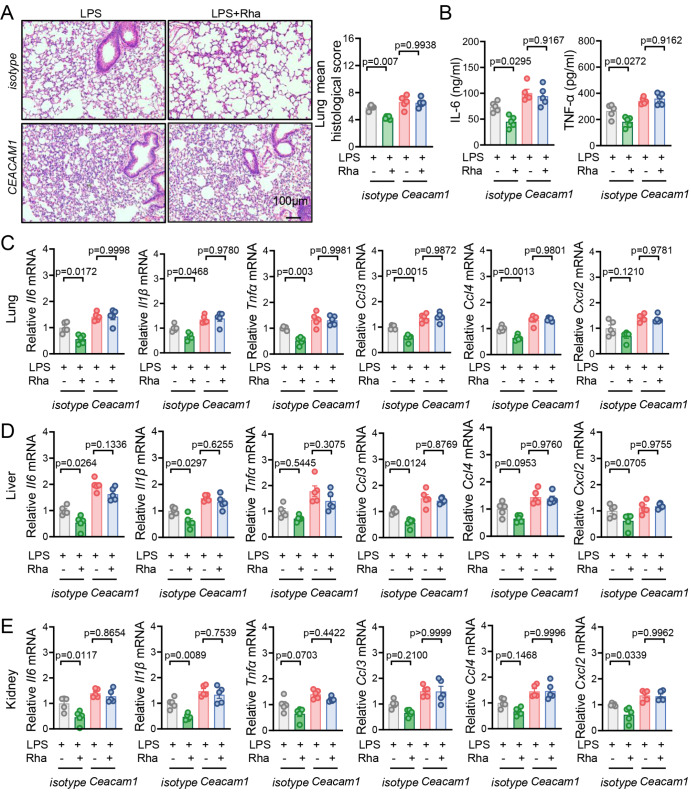
Figure 7 CEACAM1 signaling is required for rhamnose anti-inflammatory effects in LPS-induced endotoxemia mice The mice received 10 mg/kg anti-CEACAM1 antibody for one day, followed by the administration of rhamnose (74 mg/kg) or PBS prior to LPS (15 mg/kg) challenge. At 12 h post-LPS challenge, the mice were sacrificed, and tissue samples were collected for analysis. (A) Representative HE staining of lung tissues and corresponding histological scores (n = 5) (scale bar: 100 μm). (B) The protein levels of IL-6 and TNF-α in mouse plasma (n = 5). (C–E) The mRNA expression levels of key cytokines and chemokines were measured in lung, liver, and kidney tissues (n = 5). The data are shown as the mean ± SEM.

To confirm our *in vitro* experimental results, we used an anti-CEACAM1 antibody to neutralize CEACAM1 in mice and investigated the role of CEACAM1 signaling in the anti-inflammatory effects of rhamnose. Preadministration of an anti-CEACAM1 antibody inhibited the rhamnose-mediated attenuation of systemic inflammation and organ injury in LPS-stimulated mice ( Figure 7A–E). Furthermore, the lung injury scores and inflammatory cytokine levels were similar in the LPS alone or the LPS plus rhamnose-treated mice in the presence of the anti-CEACAM1 antibody ( Figure 7A–E). Additionally, rhamnose no longer affected p-P38 and DUSP1 expression in mouse peritoneal lavage fluid (PLF) cells after CEACAM1 inhibition with specific antibodies ( Supplementary Figure S10). These results suggest that rhamnose provides protection against LPS-induced endotoxicity in mice, partially through the CEACAM1 signaling.

## Discussion

**Figure 8:**
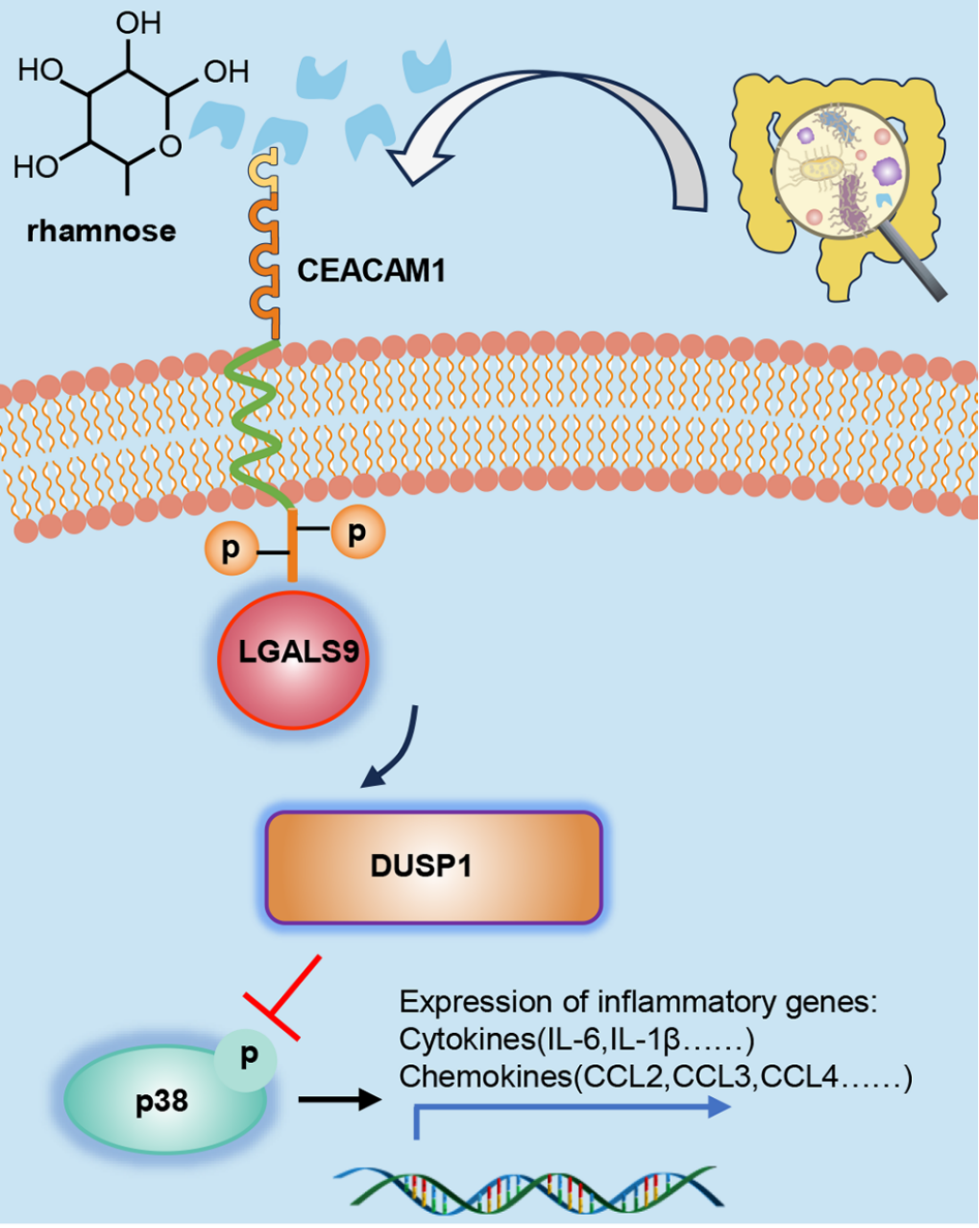
Figure 8 Working model of the effect of rhamnose on proinflammatory response Rhamnose offers protection against LPS-induced inflammation and organ damage in mice. Mechanistically, rhamnose may directly bind to CEACAM1, promoting its tyrosine phosphorylation. This phosphorylated CEACAM1 interacts with LGALS9, which enhances DUSP1 expression. As a result, the phosphorylation of p38 is inhibited, reducing the overexpression of proinflammatory factors in macrophages.

Rhamnose is a simple sugar that is not produced autonomously in host cells but can be produced by the gut microbiota [22]. Here, we report that circulating rhamnose levels are increased in endotoxemic mice. Disruption of the intestinal barrier may lead to rhamnose penetration, which may be the primary cause of elevated circulating rhamnose levels. We also determined that the microbial metabolite rhamnose can attenuate systemic inflammation and organ damage in LPS-induced endotoxemic mice. Molecular and cellular studies further demonstrated that rhamnose may functionally bind to the V39, D40, and T101 sites of CEACAM1 in macrophages and promote the interaction of CEACAM1 with LGALS9, which enhances the expression of the DUSP1 protein and inhibits the phosphorylation of p38, ultimately alleviating excessive inflammation and organ damage ( Figure 8).

The gut microbiota is essential for maintaining immune system balance in the host [ 44– 47]. Gut commensal bacteria contribute to immune training; thus, germ-free mice exhibit defective immune responses [ 48 , 49]. The metabolites of gut commensal bacteria are double-edged swords; some bacterial products can cause uncontrollable inflammation and accelerate the progression of many diseases, whereas others have anti-inflammatory properties that protect against worsening disease conditions [ 50 , 51]. Currently, developing an effective anti-inflammatory approach is necessary to control the severity of inflammation-associated diseases. However, the bioactive effects of rhamnose, a product derived from intestinal bacteria, remain unclear. Hence, the use of rhamnose in the clinic is limited. Our data revealed that rhamnose may be safe (does not cause inflammation or organ injury) for mice at a certain dose. Indeed, rhamnose was previously recognized as “nonfunctional”. However, our current evidence clearly shows that this simple sugar can serve as a safe and functional compound to suppress infectious inflammation, which encouraged us to consider the use of rhamnose in the clinic in the future.

Evidence has shown that p38 activity is critical in the inflammatory response [52]. DUSP1 is a key negative regulator of proinflammatory cytokine production through dephosphorylation of p38 [53]. Increased DUSP1 expression deactivates the p38 pathway, leading to a decrease in inflammatory factors such as IL-6, IL-1β, and TNF-α [ 54, 55]. Our study corroborates earlier findings that rhamnose upregulates DUSP1 expression and inhibits p38 activation and cytokine production after LPS stimulation.

In this study, the upstream mechanisms by which rhamnose regulates p38 in the LPS-induced inflammatory response in macrophages were also explored. Previous studies have suggested that certain membrane proteins may exist in mammalian cell membranes that directly interact with rhamnose [26], but little research has been done in this area. This study identifies CEACAM1 as a potential membrane receptor for rhamnose. CEACAM1 is a cell-surface transmembrane glycoprotein expressed on epithelial cells, neutrophils, and macrophages [56]. The *CEACAM1* gene is differentially spliced to produce functionally distinct short and long isoforms. Among them, the cytoplasmic short domain of CEACAM1 interacts primarily with the actin cytoskeleton, while the long domain features two immunotyrosine-based inhibitory motifs involved in immunosuppressive signaling [57]. Research has indicated that CEACAM1 deficiency exacerbates liver inflammation in a mouse model of autoimmune hepatitis [58]. In contrast, in liver-specific transgenic mice, CEACAM1 overexpression attenuated inflammatory injury [59]. Additionally, many gram-negative pathogens, including Neisseria, commonly utilize CEACAM1 as a cellular receptor to effectively inhibit the inflammatory response [60]. These findings suggest that CEACAM1 may be involved in the regulation of the inflammatory response. Our study confirmed the direct binding of rhamnose to CEACAM1 through various experimental methods, including DARTS, CETSA, and immunofluorescence colocalization. Interestingly, the inhibition of CEACAM1 expression enhanced inflammation-related signaling, including the downregulation of DUSP1, increased p38 phosphorylation, and increased the expressions of inflammatory factors. These findings indicate that gut microbiota-derived rhamnose protects the host from endotoxin-induced infectious inflammation, possibly by binding to its potential ‘receptor’, CEACAM1.

LGALS9, a ubiquitously expressed β-galactoside-binding lectin belonging to the tandem repeat subclass of the lectin superfamily, is widely distributed in the cytoplasm [61]. LGALS9 plays diverse biological roles, such as modulating inflammation and immunity, facilitating cell adhesion, and inducing apoptosis [ 62– 64]. Intracellular LGALS9 has been shown to activate inflammatory cytokines in monocytes [39]. In the liver tissues of chronic hepatitis B patients, the expression of LGALS9 is upregulated during the immunologically active and hyperinflammatory phases [65]. These findings indicate that LGALS9 may be involved in regulating the immune system. Our findings indicate that rhamnose enhances tyrosine phosphorylation of CEACAM1 upon LPS stimulation and promotes the interaction of CEACAM1 with LGALS9. Notably, by knocking down *LGALS9*, we discovered that the inhibitory effect of rhamnose on the inflammatory response (including the upregulation of DUSP1, attenuation of p38 phosphorylation, and reduction in inflammatory factor expression) is at least partially dependent on LGALS9 and may be associated with the tyrosine phosphorylation of CEACAM1-LGALS9. However, the exact molecular mechanisms by which CEACAM1 phosphorylation contributes to this process remain to be fully elucidated. We hypothesize that CEACAM1 phosphorylation may regulate LGALS9 activity and DUSP1 expression through the following mechanisms: (1) CEACAM1 phosphorylation may induce conformational changes or alter its binding affinity with LGALS9, thereby enhancing or stabilizing their interaction, ultimately leading to DUSP1 expression and p38 phosphorylation. (2) The phosphorylation state of CEACAM1 influences the spatial localization and intracellular distribution of LGALS9. (3) This phosphorylation may recruit or activate downstream signaling molecules (such as Src family kinases), thereby regulating the MAPK signaling pathway, which ultimately affects DUSP1 expression and p38 dephosphorylation. Previous studies have shown that LGALS9 can influence p38 phosphorylation, whereas DUSP1 is a key target of MAPK phosphorylation [ 37, 66]. On the basis of our findings, we propose that tyrosine phosphorylation of the cell surface receptor CEACAM1 may facilitate its binding to LGALS9, which could indirectly influence the expression of the p38 upstream regulator DUSP1 and consequently modulate the phosphorylation of p38. Future functional experiments are needed to clarify whether CEACAM1 functions upon phosphorylation and how its interaction with LGALS9 regulates the protein expression of DUSP1.

Together, these findings support the hypothesis that the rhamnose/CEACAM1/LGALS9-p38 axis plays a significant role in inflammation, providing valuable information for the development of novel anti-inflammatory strategies. Future studies should further clarify the mechanism of action of these molecules in the clinic and explore their potential as novel anti-inflammatory agents in combination with existing therapeutic approaches.

In this study, we focused on the role of rhamnose in endotoxin-induced infectious inflammation. Our study demonstrated that rhamnose alleviates endotoxin-induced systemic inflammation and organ damage. Rhamnose binds to CEACAM1 in macrophages, promoting the tyrosine phosphorylation of CEACAM1 and further recruiting intracellular LGALS9. This ultimately increases DUSP1 expression and p38 dephosphorylation, exerting an inhibitory effect on inflammation. The rhamnose/CEACAM1/LGALS9-p38 pathway in macrophages may be essential for host defense against infectious inflammation. Future work may focus on how to translate these findings into the clinical treatment of infection-induced inflammation.

## Supporting information

690FigS1-10-TabS1-2

## Supplementary Data

Supplementary data is available at *Acta Biochimica et Biophysica Sinica* online.
