# Supplementary material for: Rhamnose alleviates the proinflammatory response during endotoxemia via the CEACAM1/LGALS9-p38 axis: Rhamnose alleviates the proinflammatory response
Source: Acta Biochim Biophys Sin (Shanghai). 2025 Jul 24;57(12):1983–98. doi: 10.3724/abbs.2025109 (PMC12747936; doi:10.3724/abbs.2025109)
Supplement: 690FigS1-10-TabS1-2 [file 690FigS1-10-TabS1-2.docx]

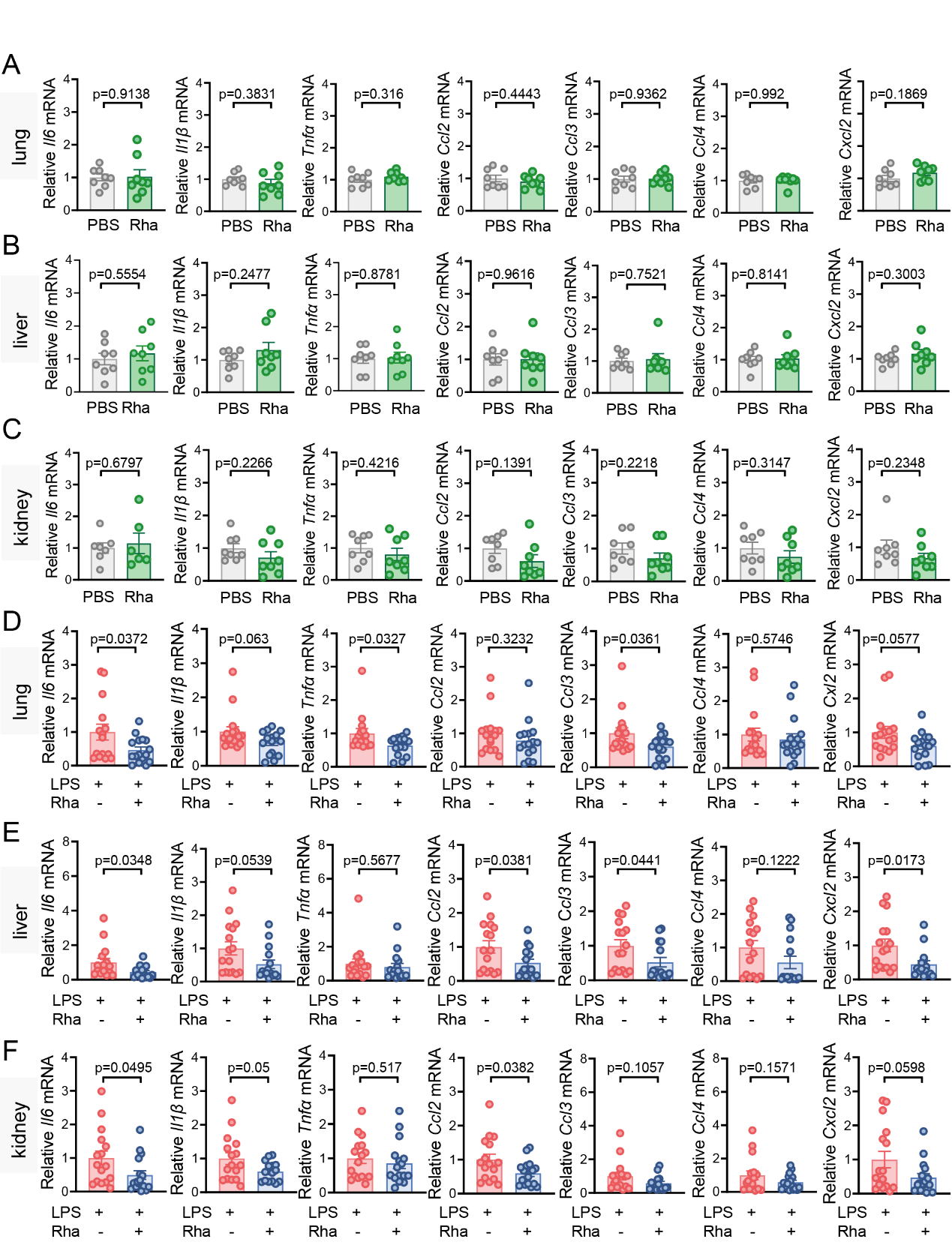


**Supplementary Figure S1. Gut bacteria-derived rhamnose protects against endotoxemia** (A−C) Mice received oral administration of PBS or rhamnose (74 mg/kg) for 12 h. The mRNA expression levels of key cytokines and chemokines were measured in the lung, liver, and kidney (*n* = 6−8). (D−F) Mice were gavaged with rhamnose (74 mg/kg) or PBS for 2 h before LPS challenge. The mRNA expression levels of key cytokines and chemokines were measured in lung, liver, and kidney tissue (*n* = 15−16). Data are shown as the mean ± SEM.


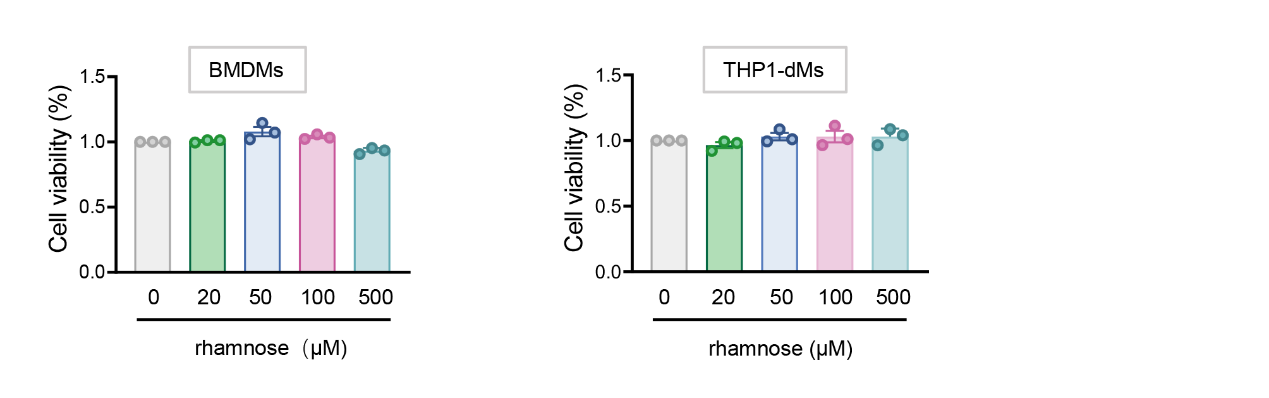


**Supplementary Figure S2. Dose safety evaluation of rhamnose *in vitro*** After 6 h of incubation with different doses of rhamnose, the cell viability of BMDMs and THP1-dMs was examined by Cell Counting Kit-8 (CCK8) assay (*n* = 3). Data are shown as the mean ± SEM.


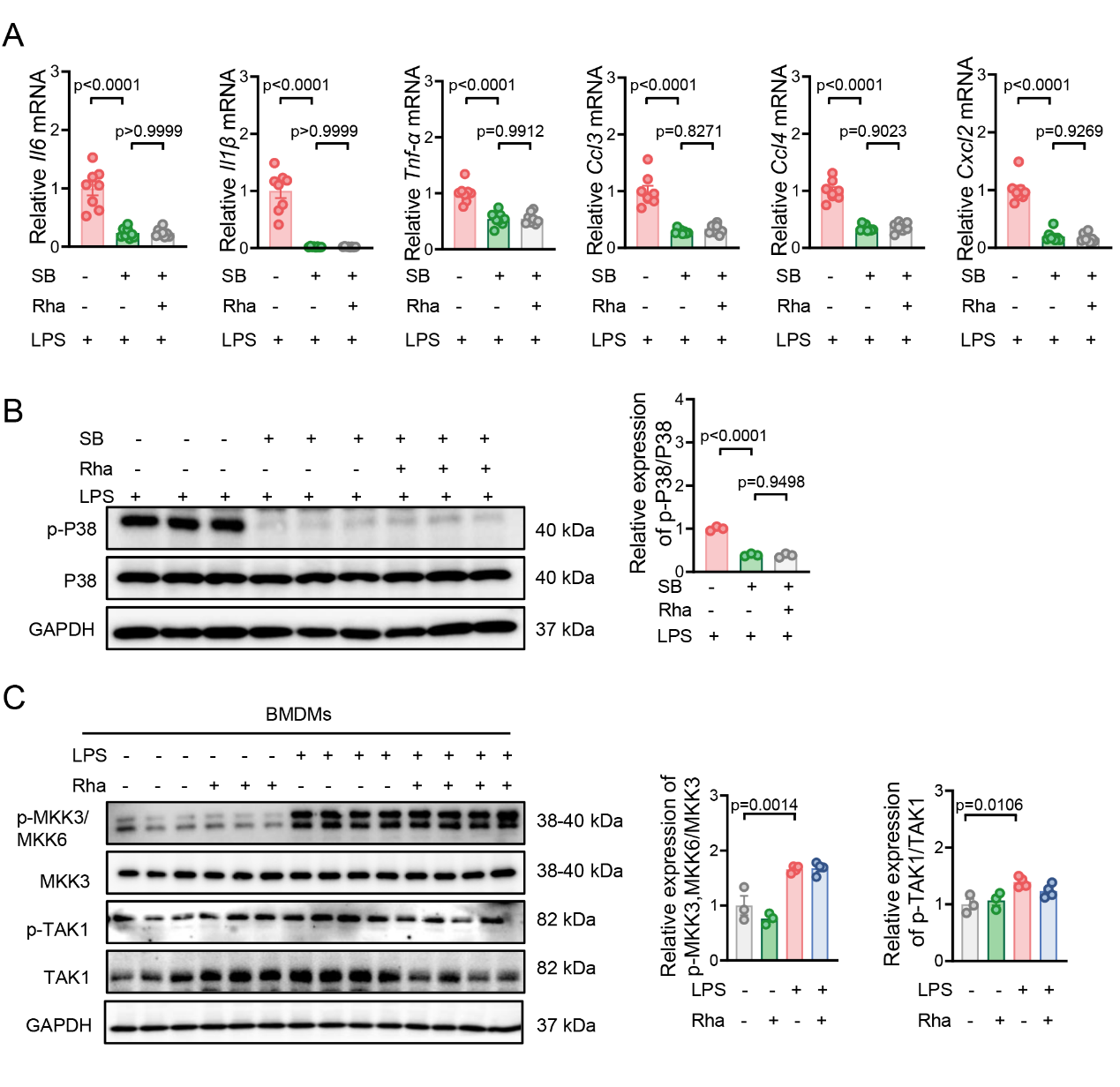


**Supplementary Figure S3. The anti-inflammatory effect of rhamnose partially relies on the p38 MAPK pathway** (A) The THP1-dMs were pretreated with or without SB203580 (10 μM) for 1 h, followed by a 6-h incubation with LPS (500 ng/mL) and rhamnose (20 μM). The mRNA expression levels of major cytokines and chemokines were measured (*n* = 8). (B) The THP1-dMs were pretreated with or without SB203580 (10 μM) for 1 h, followed by a 15-min incubation with LPS (500 ng/mL) and rhamnose (20 μM). Western blot analysis of p-P38, total P38, and GAPDH levels (*n* = 3). (C) Western blot analysis of P-MKK3/MKK6, MKK3, p-TAK1, TAK1, and GAPDH levels in BMDMs following a 15-min LPS stimulation (100 ng/mL), with or without rhamnose (20 μM) treatment (*n* = 3−4). Data are shown as the mean ± SEM.


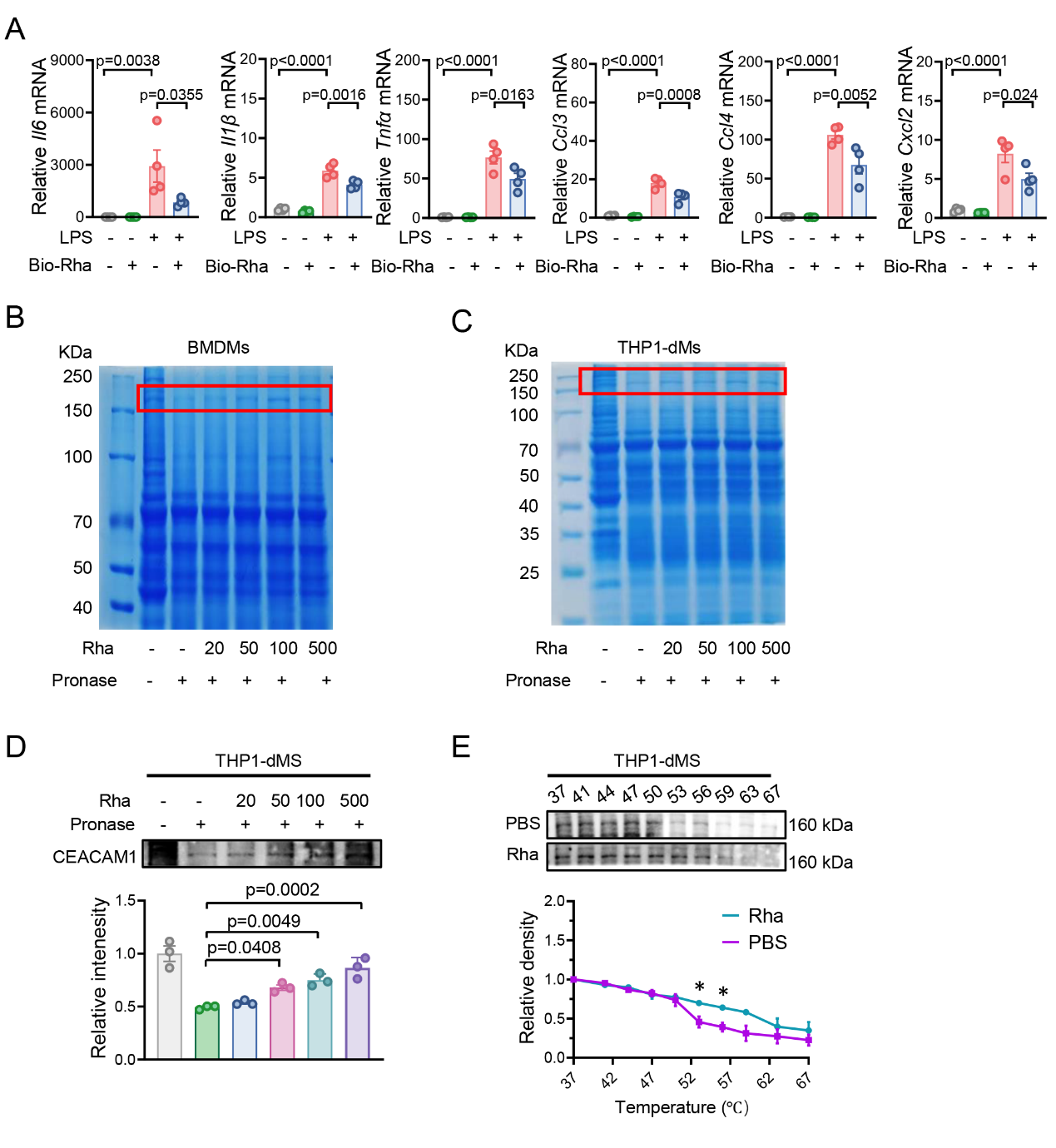


**Supplementary Figure S4. Identification of a target protein of rhamnose** (A) The mRNA expression of pro-inflammatory factors was measured in THP1-dMs following a 6-h LPS (500 ng/mL) stimulation, with or without biotin-labeled rhamnose (Bio-Rha, 20 μM) (*n* = 4). (B,C) Coomassie blue staining showed a marked increase in the 150−250 kDa band when incubated with rhamnose in pronase-digested BMDMs (B) or THP1-dMs (C) lysates. (D) Western blot analysis of CEACAM1 degradation in pronase-digested lysates from THP1-dMs cells, with and without rhamnose (*n* = 3). (E) The CETSA analysis of CEACAM1 degradation in THP1-dMs lysates with or without rhamnose (100 µM) treatment for 2 h (*n* = 3). Data are shown as the mean ± SEM.


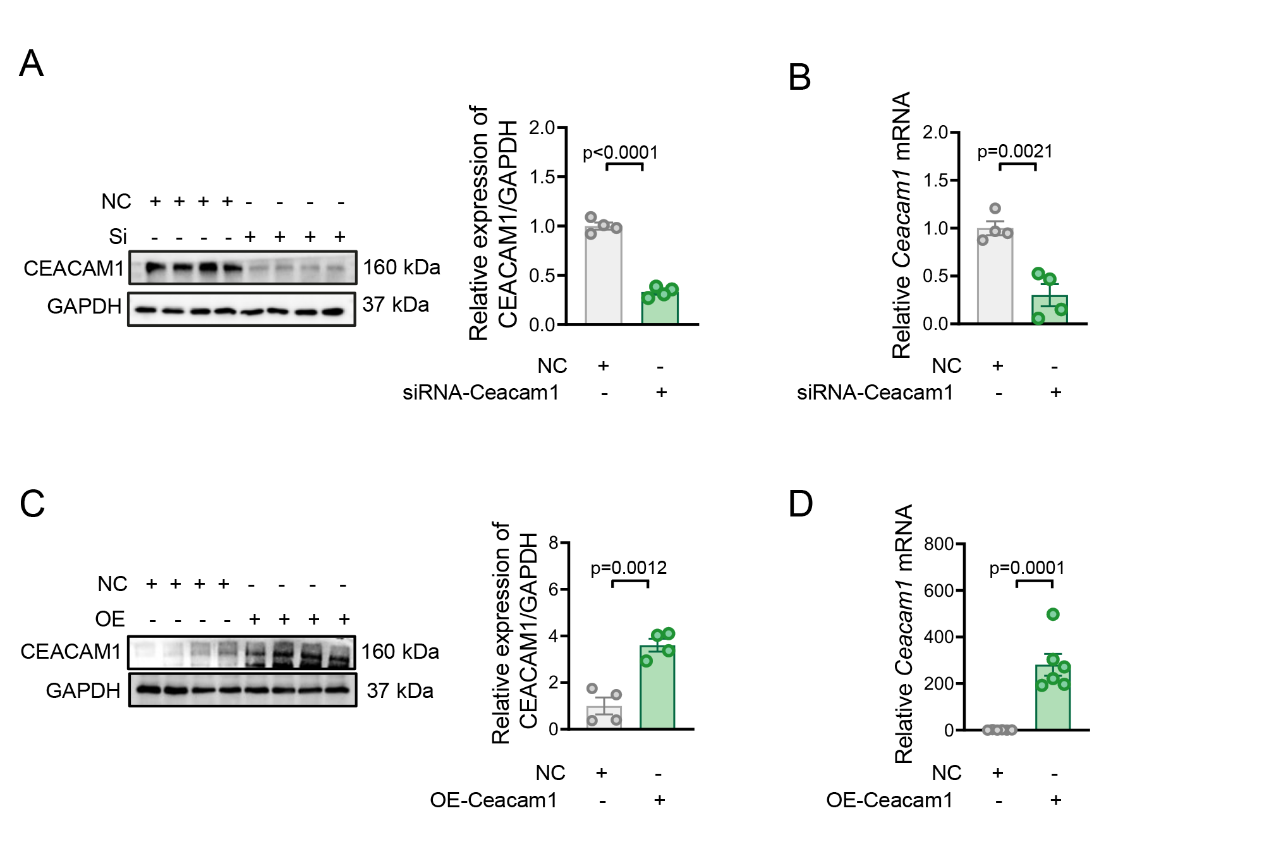


**Supplementary Figure S5. Transfection efficiency of CEACAM1** (A) Western blot analysis of CEACAM1 and GAPDH levels in THP1-dMs after transfection with CEACAM1 or control siRNA for 48 h (*n* = 4). (B) The mRNA levels of *CEACAM1* were measured in THP1-dMs after transfection with CEACAM1 or control siRNA for 48 h (*n* = 4). (C) Western blot analysis of CEACAM1 and GAPDH levels in THP1-dMs after transfection with control plasmid or CEACAM1-overexpression plasmid (CEACAM1-OE) for 48 h (*n* = 4). (D) The mRNA levels of *CEACAM1* were measured in THP1-dMs after transfection with control plasmid or CEACAM1-overexpression plasmid (CEACAM1-OE) for 48 h (*n* = 6). Data are shown as the mean ± SEM.


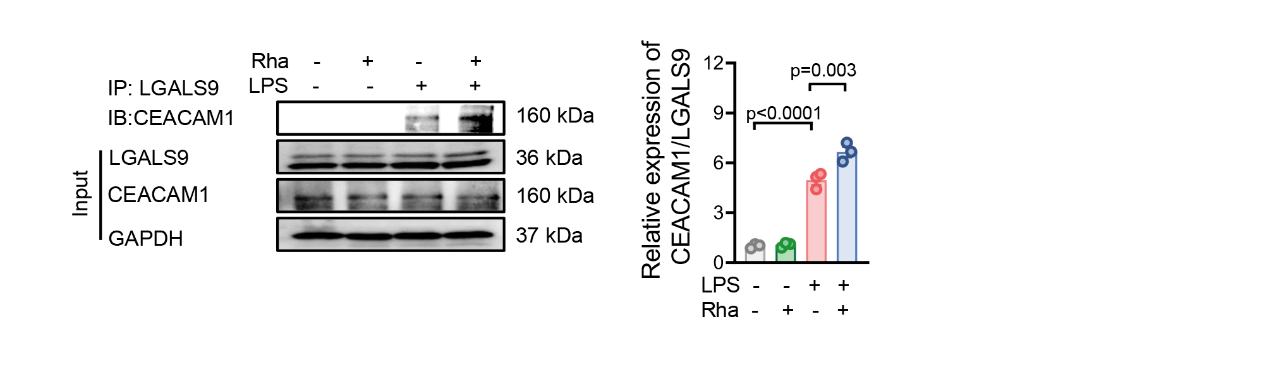


**Supplementary Figure S6. Rhamnose promotes the binding of CEACAM1 to LGALS9** Western blot analysis of LGALS9, CEACAM1, and GAPDH levels. THP1-dMs were exposed to LPS (500 ng/mL) for 15 min, with or without rhamnose (20 μM), followed by immunoprecipitation using an anti-LGALS9 antibody (*n* = 3). Data are shown as the mean ± SEM.


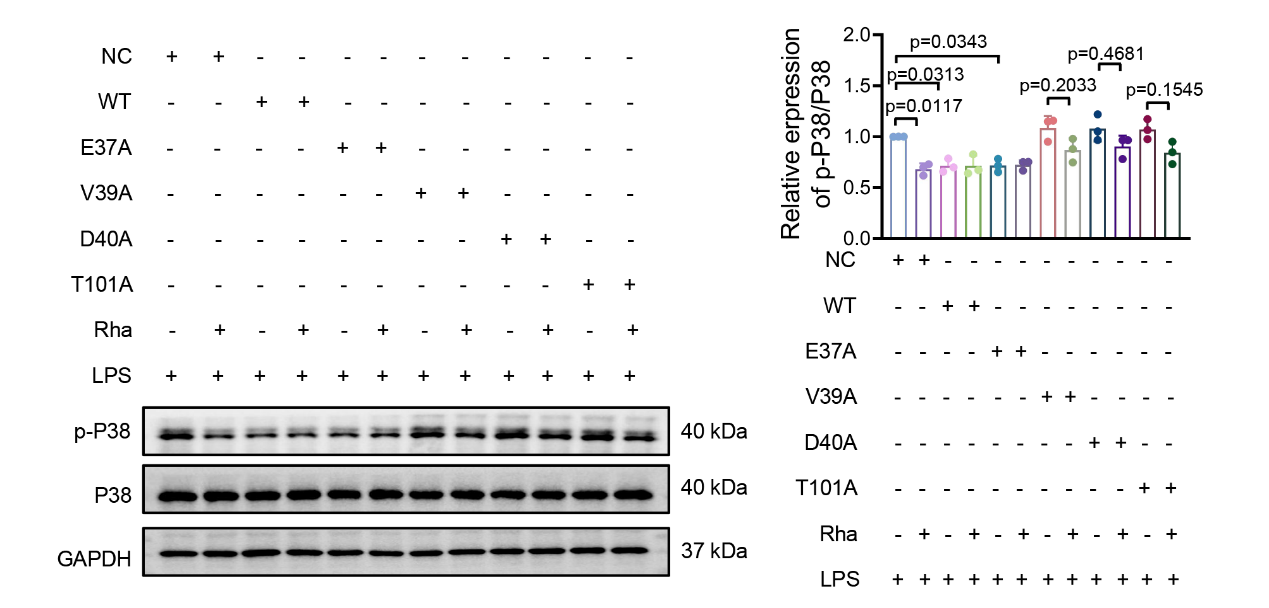


**Supplementary Figure S7. The effects of wild-type (WT) and mutant CEACAM1 on downstream p-p38 expression**  THP1-dMs were co-transfected with WT-Flag-CEACAM1 or different Flag-CEACAM1 mutants (E37A, V39A, D40A, or T101A) and stimulated with LPS (500 ng/mL) in the presence or absence of rhamnose (20 μM). Western blot analysis of p-P38, total P38, and GAPDH levels (*n* = 3). Data are shown as the mean ± SEM.


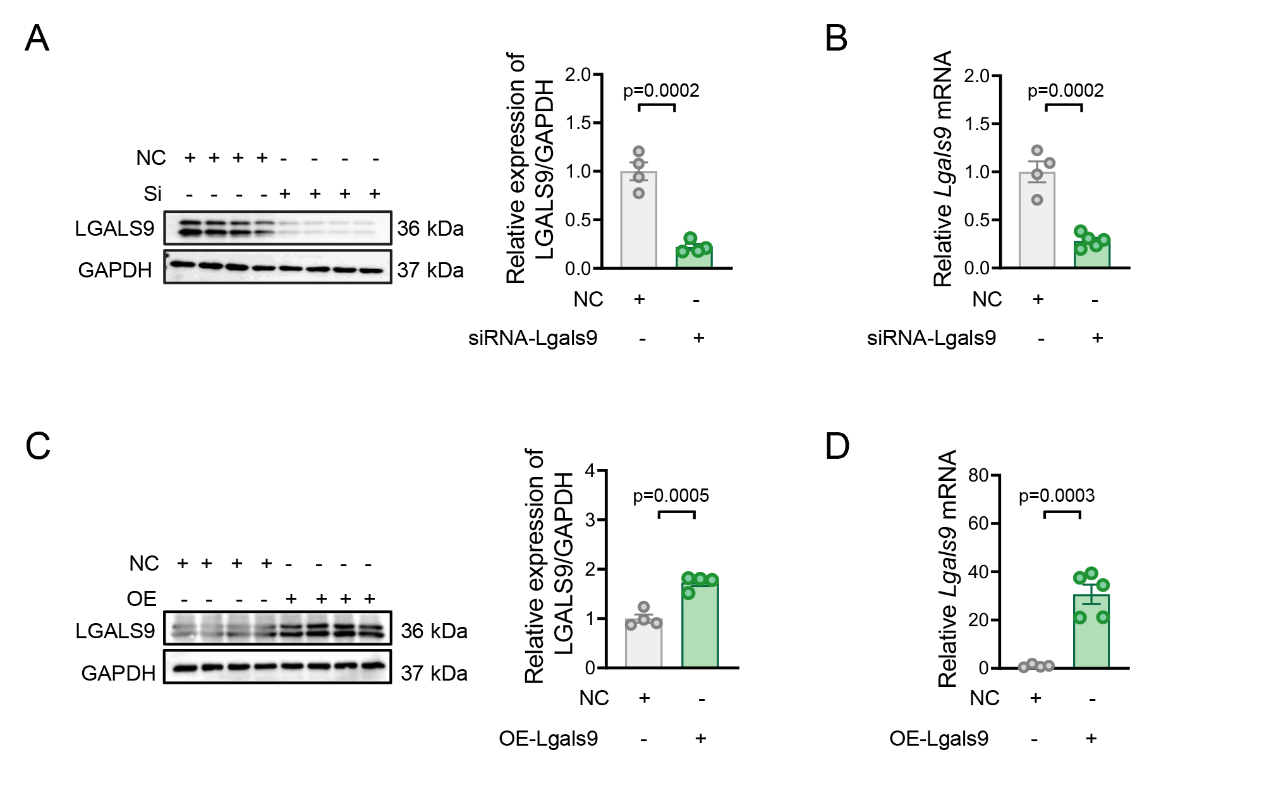


**Supplementary Figure S8. Transfection efficiency of LGALS9** (A) Western blot analysis of LGALS9 and GAPDH levels in THP1-dMs after transfection with LGALS9 or control siRNA for 48 h (*n* = 4). (B) The mRNA levels of *LGALS9* were measured in THP1-dMs after transfection with LGALS9 or control siRNA for 48 h (*n* = 4−5). (C) Western blot analysis of LGALS9 and GAPDH levels in THP1-dMs after transfection with control plasmid or LGALS9-overexpression plasmid (LGALS9-OE) for 48 h (*n* = 4). (D) The mRNA levels of *LGALS9* were measured in THP1-dMs after transfection with control plasmid or LGALS9-overexpression plasmid (LGALS9-OE) for 48 h (*n* = 4−5). Data are shown as the mean ± SEM.


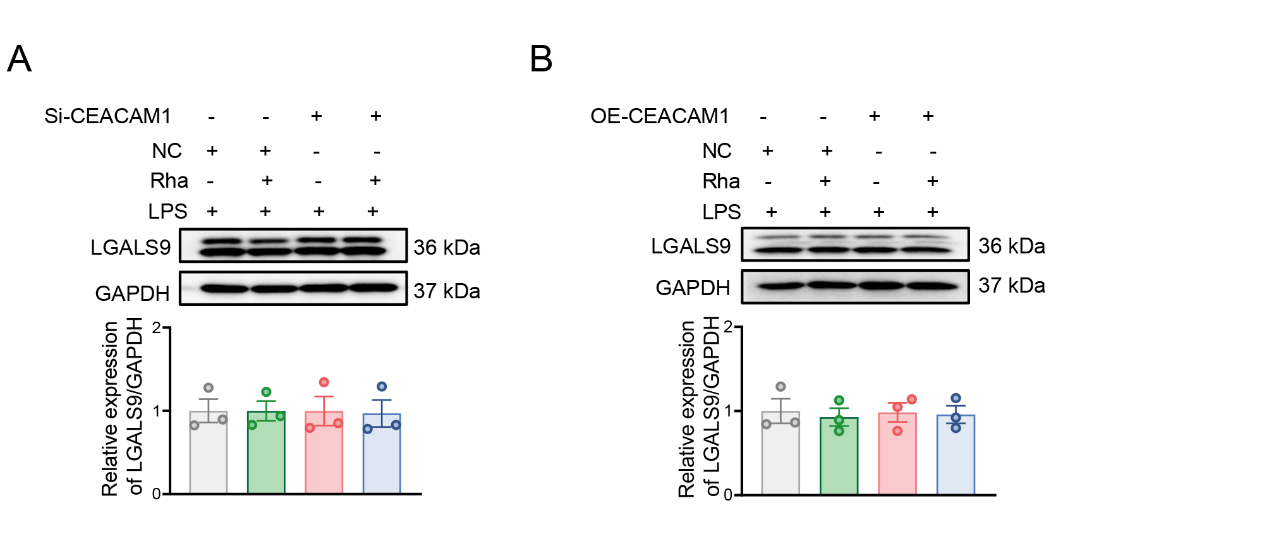


**Supplementary Figure S9. CEACAM1 does not affect the expression of LGALS9** (A) THP1-dMs were transfected with CEACAM1 or control siRNA for 48 h, followed by a 15-min incubation with LPS (500 ng/mL), with or without rhamnose (20 μM). Western blot analysis of LGALS9 and GAPDH levels (*n* = 3). (B) THP1-dMs were transfected with empty plasmid or CEACAM1-overexpression plasmid (CEACAM1-OE) for 48 h, followed by a-15 min incubation with LPS (500 ng/mL), with or without rhamnose (20 μM). Western blot analysis of LGALS9 and GAPDH levels (*n* = 3). Data are shown as the mean ± SEM.


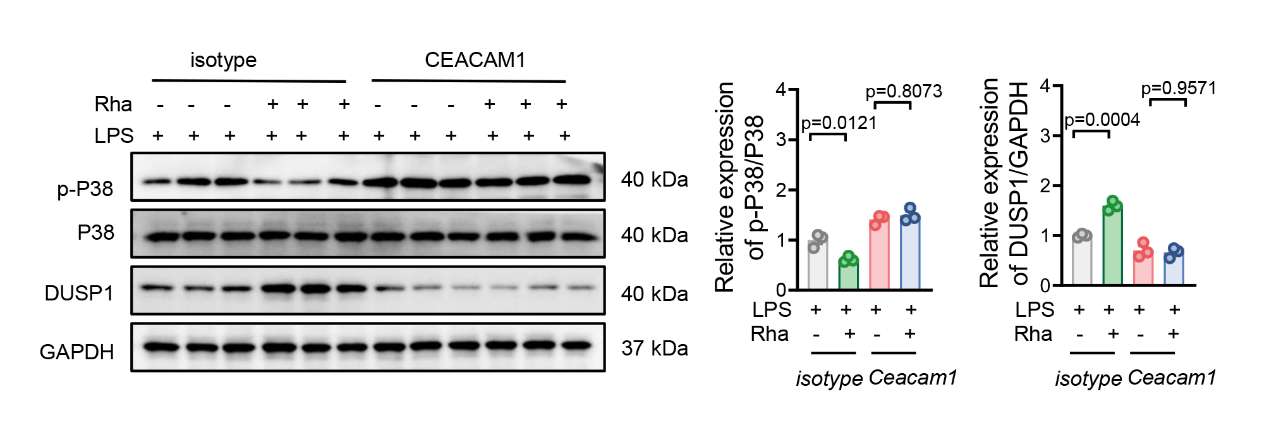


**Supplementary Figure S10.** **Rhamnose pretreatment does not alter the expressions of p-P38 and DUSP1 in peritoneal lavage fluid cells of CEACAM1-neutralised mice** Mice received a 10 mg/kg dose of anti-CEACAM1 antibody for one day, followed by the administration of rhamnose (74mg/kg) or PBS prior to LPS (15 mg/kg) challenge. The mice were sacrificed at 12 h after LPS challenge and the peritoneal fluid cells were collected for further analysis. Western blot analysis of mouse peritoneal lavage fluid cells to assess p-P38, total P38, DUSP1, and GAPDH levels (*n* = 3). Data are shown as the mean ± SEM.

**Supplementary Table S1. Information of antibodies used in this study**

| Antibodies | Source | Cat# | Dilution |
| --- | --- | --- | --- |
| TAK1 | Cell Signaling Technology | 5206S | 1:1000 |
| p-TAK1 | Cell Signaling Technology | 9339S | 1:1000 |
| JNK | Cell Signaling Technology | 9252S | 1:1000 |
| p-JNK | Cell Signaling Technology | 4668S | 1:1000 |
| ERK | Cell Signaling Technology | 4695S | 1:1000 |
| p-ERK | Cell Signaling Technology | 4370S | 1:1000 |
| P38 | Cell Signaling Technology | 8690S | 1:1000 |
| p-P38 | Cell Signaling Technology | 9211S | 1:1000 |
| MKK3 | Cell Signaling Technology | 8535S | 1:1000 |
| p-MKK3/MKK6 | Cell Signaling Technology | 9236S | 1:1000 |
| CEACAM1 | Abcam | 108397 | 1:1000 |
| LGALS9 | Cell Signaling Technology | 54330S | 1:1000 |
| DUSP1 | Cell Signaling Technology | 35217S | 1:1000 |
| Anti-phosphotyrosine | Merck | 05-321 | 1:1000 |

**Supplementary Table S2. Sequences of primers used for qPCR in this study**

| Gene | Forward primer (5′→3′) | | Reverse primer (5′→3′) |
| --- | --- | --- | --- |
| Mouse |  | |  |
| *18S* | AGTCCCTGCCCTTTGTACACA | | CGATCCGAGGGCCTCACTA |
| *IL6* | ACCAGAGGAAATTTTCAATAGGC | | TGATGCACTTGCAGAAAACA |
| *IL-1β* | GGTCAAAGGTTTGGAAGCAG | | TGTGAAATGCCACCTTTTGA |
| *Tnf-α* | AGGGTCTGGGCCATAGAACT | | CCACCACGCTCTTCTGTCTAC |
| *Ccl2* | ATTGGGATCATCTTGCTGGT | | CCTGCTGTTCACAGTTGCC |
| *Ccl3* | GTGGAATCTTCCGGCTGTAG | | ACCATGACACTCTGCAACCA |
| *Ccl4* | GAAACAGCAGGAAGTGGGAG | | CATGAAGCTCTGCGTGTCTG |
| *Cxcl2* | TCCAGGTCAGTTAGCCTTGC | | CGGTCAAAAAGTTTGCCTTG |
| Human |  | |  |
| *18S* | | AGGAATTCCCAGTAAGTGCG | GCCTCACTAAACCATCCAA |
| *IL6* | | CGGGAACGAAAGAGAAGCTC | ACAACAACAATCTGAGGTGC |
| *IL-1β* | | AATCTGTACCTGTCCTGCGTGTT | TGGGTAATTTTTGGGATCTACACT |
| *Tnf-α* | | CTCCCAGGTCCTCTTCAAGG | TTGATGGCAGAGAGGAGGTT |
| *Ccl3* | | ACTTTGAGACGAGCAGCCAGTG | TTTCTGGACCCACTCCTCACTG |
| *Ccl4* | | GCTTCCTCGCAACTTTGTGGTAG | GGTCATACACGTACTCCTGGAC |
| *Cxcl2*  *Ceacam1*  *Lgals9* | | TGTCTCAACCCCGCATCG  CCTGGCTTATCAATGGAACA  GGACGGACTTCAGATCACTGT | AGGAACAGCCACCAATAAGC  ACTGAGTTATTGGCGTGGC  CCATCTTCAAACCGAGGGTTG |
